# Elucidation of the *ty-5* resistance network in tomato against tomato yellow leaf curl virus reveals the involvement of AP2/ERF gene

**DOI:** 10.3389/fpls.2026.1788099

**Published:** 2026-04-15

**Authors:** Chellappan Padmanabhan, Qiyue Ma, Md Shamimuzzaman, Kevin S. Stewart, Jennifer R. Wilson, Daniel K. Hasegawa, Reza Shekasteband, Samuel F. Hutton, John W. Scott, Alvin M. Simmons, Zhangjun Fei, Kai-Shu Ling

**Affiliations:** 1 U.S. Vegetable Laboratory, U.S. Department of Agriculture - Agricultural Research Service, Charleston, SC, United States; 2 Boyce Thompson Institute, Cornell University, Ithaca, NY, United States; 3 Agroecosystem Management Research Unit, U.S. Department of Agriculture - Agricultural Research Service, Lincoln, NE, United States; 4 Gulf Coast Research and Education Center, Institute of Food and Agricultural Sciences, University of Florida, Wimauma, FL, United States

**Keywords:** APETALA2/ethylene responsive factor, DNA methylation, RNA-dependent RNA polymerase, tomato yellow leaf curl virus, transcriptome profiling, *ty-5* resistance

## Abstract

**Introduction:**

Tomato yellow leaf curl virus (TYLCV, *Begomovirus coheni*), a whitefly-transmitted begomovirus, causes serious economic losses to tomato crops globally. Over the past three decades, several genetic sources of TYLCV resistance have been identified and incorporated into tomato breeding. Among these is a recessive source of resistance known as *ty-5*.

**Methods:**

In this study, we conducted a global transcriptome analysis to compare gene expression in a *ty-5* near isogenic tomato line and its susceptible recurrent parent at various stages of TYLCV infection.

**Results:**

We identified 1,394 differentially expressed genes, including numerous defense-related genes and genes involved in RNA-mediated DNA methylation. In addition, the expression of key regulatory genes such as protein kinases (PKs) and transcription factors (TFs) was also significantly altered. To explore the function of one upregulated TF encoding an APETALA2/Ethylene Responsive Factor (AP2/ERF), tomato plants overexpressing this gene were generated and demonstrated to confer resistance to TYLCV.

**Discussion:**

These results lead us to hypothesize that *ty-5*-mediated TYLCV resistance not only involves a *pelota* gene but is a product of two major pathways acting in concert: a typical host-disease resistance pathway and RNA-directed DNA methylation. Understanding the mechanism underlying *ty-5* resistance will facilitate the development of tomato germplasm with more durable TYLCV resistance.

## Introduction

Tomato (*Solanum lycopersicum* L.) is one of the world’s most important vegetable crops. Viral diseases are a major limiting factor in tomato production (Hanssen et al., 2010; Hančinský et al., 2020). tomato yellow leaf curl virus (TYLCV, *Begomovirus coheni*), a whitefly (*Bemisia tabaci*)-transmitted begomovirus first identified in the Middle East in 1959 (Cohen et al., 1963), has caused serious economic losses (up to 100%) to tomato crops in the U.S. and other parts of the world (Lefeuvre et al., 2010; Scholthof et al., 2011; Prasad et al., 2020; Yan et al., 2021). TYLCV is a DNA virus in the genus *Begomovirus* under the family *Geminiviridae*. This virus consists of a single-stranded circular DNA with 2787 nt encoding six open reading frames (ORF): two (V1 and V2) in the sense orientation and four others (C1 to C4) in the complementary orientation. V1 encodes coat protein to protect the viral DNA. V2 is a pre-coat protein, associated with viral movement. C1, C2, C3, and C4 proteins are for virus replication, post-transcriptional gene silencing suppressor, virus accumulation enhancer and symptom induction determinant, respectively (Glick et al., 2009). Recently, several small proteins (V3, C5 and C7) have also been identified in TYLCV with the following functions, an RNA silencing suppressor, a virulence factor and suppressor of gene silencing, and a pathogenicity determinant, respectively (Gong et al., 2021; Zhao et al., 2022; Liu et al., 2023).

TYLCV is transmitted efficiently by an insect vector, the whitefly (*Bemisia tabaci*) in a persistent-circulative nonpropagative matter. *B. tabaci* is a species complex consisting of 34 cryptic species with similar morphology but distinct genetic and biological properties (Boykin and De Barro, 2014). Several of them, including Mediterranean (MED) and Middle East-Asia minor 1 (MEAM1) have become global pests with efficient transmission of hundreds of viruses, including TYLCV (Navas-Castillo et al., 2011). An integrated pest management system focusing on prevention, control and resistance would be necessary to effectively manage *B. tabaci* and TYLCV transmission (Prasad et al., 2020).

Resistance to TYLCV has been discovered in several wild tomato species, including *Solanum pimpinellifolium*, *S. peruvianum*, *S. chilense*, *S. habrochaites*, and *S. cheesmaniae* (Pico et al., 1996; 2000; Ji et al., 2007; 2009). To date, six TYLCV resistance loci have been identified in various species of tomato (El-Sappah et al., 2022): *Ty-1*, introgressed from *S. chilense* accession LA1969 and mapped to chromosome 6 (Zamir et al., 1994); *Ty-2*, derived from *S. habrochaites* accession B6013 and located on chromosome 11 (Kalloo and Banerjee, 1990; Hanson et al., 2006); *Ty-3*, from *S. chilense* accessions LA1932 and LA2779 and mapped to chromosome 6, near the *Ty-1* locus (Ji et al., 2007); *Ty-4*, from *S. chilense* accession LA1932 and mapped to chromosome 3 (Ji et al., 2009); the recessive *ty-5* gene, derived from the cultivar Tyking and mapped to chromosome 4 (Anbinder et al., 2009; Hutton et al., 2012); *Ty-6*, also derived from ‘Tyking’ and mapped to chromosome 10 (Gill et al., 2019; Shen et al., 2025). Genes underlying the *Ty-1* and *Ty-3* loci were identified and found to be two different alleles of the same gene (Caro et al., 2015), which codes for an RNA-dependent RNA polymerase (RDRP) (Verlaan et al., 2013) that confers resistance through cytosine methylation of the viral DNA genome (Butterbach et al., 2014). The underlying genes for other Ty genes (*Ty-2*, *ty-5* and *Ty-6*) have also been characterized (Yamaguchi et al., 2018; Shen et al., 2020; Lapidot et al., 2015; Shen et al., 2025). PCR-based markers tightly linked to *Ty-1, Ty-2, Ty-3, Ty-4*, and *ty-5* are being used in tomato breeding programs for efficient selection of begomovirus resistance (El-Sappah et al., 2022).

Initial work on mapping the *ty-5* resistance gene identified *NAC Domain 1* (*NAC1*) at the *ty-5* locus (Anbinder et al., 2009). However, subsequent fine-mapping by Lapidot et al. (2015) revealed that the *ty-5* resistance gene encodes pelota (Pelo), a messenger RNA surveillance factor. Knockdown experiments confirmed that *Pelo* was responsible for a recessive resistance to TYLCV through a natural mutation to pelota (Lapidot et al., 2015). In mRNA surveillance, Pelota exhibits a dual function (Li et al., 2025). In addition to maintaining translational fidelity, pelota could serve either as a restriction or a promotion factor for virus propagation depending on the species of the virus and its host (Li et al., 2025). Despite those efforts, information on molecular mechanism underlying the *ty-5* tomato plant upon TYLCV infection is still unknown.

RNA sequencing (RNA-seq) technology has been used to investigate the molecular basis of TYLCV resistance in tomato. For example, Chen et al. (2013) examined differentially expressed defense-related genes associated with *Ty-2*-mediated resistance during early stages of TYLCV infection. Wang et al. (2022) evaluated differential gene expression in TYLCV-infected tomato plants upon pretreatment with hormones. An integration of transcriptome, sRNAome and methylome was also applied to evaluate viral gene expression, vsRNA accumulation and DNA methylation on TYLCV (Piedra-Aguilera et al., 2019). Transcriptome analysis revealed networks of genes in vector whiteflies *Bemisia tabaci*) were activated upon TYLCV feeding and transmission (Hasegawa et al., 2018). Currently, there is a lack of information regarding the molecular mechanism that regulates the resistance to TYLCV on tomato plants expressing the recessive *ty-5* gene. Here, we report a comparative analysis of global gene expression between a near-isogenic line carrying the *ty-5* resistance gene and the TYLCV-susceptible recurrent parent (Fla. 8059) in response to TYLCV infection. In total, 1,394 differentially expressed genes (DEGs) were identified between the two lines across four timepoints, spanning from virus inoculation to disease symptom expression. Functional category analysis revealed that two networks of genes were most affected: those involved in resistance gene-dependent host defense and RNA-directed DNA methylation. In addition, the expression of protein kinases (PKs), transcription factors (TFs), and microRNA target genes, which are important regulators of gene expression, was also altered. To evaluate gene function, transgenic tomato plants overexpressing an APETALA2/Ethylene Responsive Factor (AP2/ERF) TF, an upregulated disease resistance signaling molecule identified in the RNA-seq analysis, exhibited partial TYLCV resistance. Our global transcriptome analysis provides valuable insights into the molecular mechanisms underlying ty-5-mediated TYLCV resistance, including potential networks of defense-related genes against TYLCV in *ty-5* tomato lines.

## Materials and methods

### Generation of near-isogenic lines with and without the *ty-5* gene through backcrossing to Fla. 8059

To generate resistant and susceptible tomato lines with a similar genetic background for transcriptome analysis, near isogenic lines (NILs) carrying the *ty-5* gene for resistance to TYLCV were developed. A traditional backcrossing approach was used to introgress the *ty-5* gene from ‘Tyking’ into the susceptible recurrent parent, Fla. 8059. Tightly linked molecular markers were used to select genes during the process. Backcrossing for the *ty-5* line was completed to the BC_5_ generation (theoretically 96.875% identical to the recurrent parent). These materials were used in disease resistance screening through viruliferous whitefly inoculation and for transcriptome analysis upon virus inoculation throughout the initial disease developmental stages.

### Inoculation of tomato plants through whitefly feeding

A whitefly colony (*Bemisia tabaci* MEAM-1, formerly B biotype) was collected in Charleston, SC, USA, in 2013, and maintained under controlled greenhouse conditions on collards (*Brassica oleracea*, a non-host for TYLCV). A TYLCV isolate (Ling et al., 2006) maintained on tomato cultivar ‘Moneymaker’ was used as an inoculum source for whitefly feeding. The TYLCV resistant NIL line carrying *ty-5* and its respective susceptible recurrent parental breeding line (Fla. 8059) were germinated and maintained in an insect-free BugDorm (MegaView Science Co., Taiwan) (60 x 60 x 60 cm) inside a greenhouse under natural sunlight with a temperature around 25 °C.

To acquire TYLCV, adult non-viruliferous whiteflies were transferred onto TYLCV-infected tomato plants in a BugDorm for a 2-day acquisition access period. The viruliferous whiteflies (500) were then collected and transferred to tomato plants at the two-leaf stage in a BugDorm for feeding in a 3-day inoculation access period. After 3 days, the whiteflies were killed with a spray of the insecticide imidacloprid on the test tomato plants.

The virus-inoculated plants were maintained and monitored for symptom expressions up to 21 days post inoculation (dpi) when the leaf curl symptoms on the susceptible plants had appeared. TYLCV-inoculated leaf tissues were collected (500 mg/plant) from three test plants serving as individual biological replicates for each resistant or susceptible group at each time interval: 4, 7, 14, and 21 dpi. The collected tissue samples were processed immediately or stored at -80 °C until use for total RNA extraction and RNA-seq.

### Generation of gene construct

RNAseq data analysis revealed that several genes were differentially expressed. Among them, the APETALA2/Ethylene Responsive Factor (AP2/ERF TF) gene was selected for functional evaluation on its contribution to disease resistance against TYLCV. A codon optimized synthetic gene (AP2/ERF TF) was synthesized (IDT, Coralville, IA) and inserted into pENTR-D TOPO vector and transformed into Top 10 Chemically Competent cells (Invitrogen, USA). Plasmid DNA with inserts from selected colonies were confirmed through Sanger sequencing. Construct was recombined with Gateway vector PEG202 using clonase (Invitrogen, USA) between the Cauliflower mosaic virus (CaMV) 35S promoter and nopaline synthase (NOS) terminator. The sequence confirmed AP2/ERF TF and GFP inserted binary vectors were mobilized into *Agrobacterium tumefaciens* strain LBA4404 by electroporation and selected on YM agar containing kanamycin for PEG202 selection and Streptomycin for *Agrobacterium* (Padmanabhan et al., 2019).

### *Agrobacterium*-mediated transformation and confirmation of transgenic plants

We followed an efficient protocol for tomato transformation and selection of transgenic plants as previously described (Van Eck et al., 2019). The transgenic plants were self-pollinated to generate the T1 generation. The T1 seeds were germinated on MS basal medium containing 1 mg/L Phosphinotricin for selection. Surviving germinated seedlings were transferred to pots containing sterile soil and maintained in a glasshouse at 28-29 °C and 80-90% relative humidity. Transgene insertion was confirmed by gene specific PCR and gene expression was confirmed by RT-PCR using a FLAG specific forward primer (KL17–151 FLAG-F: 5’-GACTACAAAGACGATGACGACA-3’) and ERF specific reverse primer (KL14–403 ERF-1R: 5’ ACTTCTTCCATTCATCTCGAATGTG – 3’). For the internal control, an actin primer pair was used (forward primer KL17-071 03g078400F: 5’-TTGCTGGTCGTGACCTTACT-3’ and reverse primer KL17-072 03g078400R: 5’-TGCTCCTAGCGGTTTCAAGT-3’).

### Evaluation of APETALA2/ethylene responsive factor (AP2/ERF TF) transgenic tomato plants for resistance to TYLCV

To evaluate transgenic plants over-expressing the APETALA2/Ethylene Responsive Factor (AP2/ERF TF) gene ID Solyc04g007000 for their resistance against TYLCV, five rooted plants (in 4–5 leaf stage) were regenerated. PCR-confirmed AP2/ERF TF transgenic T_0_ lines, along with similarly developed transgenic ‘Moneymaker’ plants expressing GFP, and *ty-5* resistant plants were inoculated with TYLCV-viruliferous whiteflies using the same method as described above. In addition to observing symptom expressions on the inoculated plants, virus presence in systemic leaves of each of the test plants was also measured by conventional PCR for TYLCV (Padmanabhan et al., 2022) using DNA preparations generated from leaf tissue samples collected at 7, 14, 21, and 28 dpi. Disease severity index was calculated based on symptomatic plants over total test plants, with 0: no symptom; 1 = mild mosaic on leaves; 2 = moderate mosaic on leaves; 3 = chlorotic and mild leaf curl; 4 = severe yellowing and leaf curl; and 5 = plant death (Lapidot et al., 2006).

### RNA extraction

Total RNA was extracted using TRIzol reagents with modifications. We used 500 mg of freshly collected leaf tissue in individually labeled plastic bags which were processed using a Homex-6 homogenizer (BioReba, Switzerland) with 2.25 ml of TRIzol^®^ reagent (Thermal Fisher Scientific, USA). The resulting tissue extract was transferred to a 2 ml tube, vortexed well and left at room temperature for 5 minutes (min). After spinning at 12,000 g for 10 min, 1 ml supernatant was transferred to a new Eppendorf tube, 0.4 ml of chloroform was added, and the mixture was shaken vigorously by hand for 15 seconds and left at room temp for 2–15 min to settle. The tube was centrifuged at 12,000 g for 15 min at 4°C. Approximately 600 μl of supernatant was transferred to a new tube, 1.0 ml isopropanol was added, and tubes were mixed gently by hand and left at RT for 10 min. An RNA pellet was obtained by centrifugation at 12,000 g for 10 min at 4 °C, washed twice in 70-80% ethanol, and finally dissolved in 200 μl RNase-free water. For DNase I treatment, in an Eppendorf tube with 100 μL RNA (with a total yield of 50 μg), 12 μl of 10X reaction buffer and 4 μl DNase I (Invitrogen, USA) were incubated at 37 °C for 30 min. The treated RNA solution was processed with a one-step extraction phenol/chloroform (1:1) solution. Treated RNA was precipitated after ethanol treatment and centrifuged at 12,000 g for 20 min. The RNA pellet was washed once with 75% ethanol, dried, and resuspended in nanopure water. The concentration of the resulting RNA preparation was measured with a NanoDrop spectrophotometer. The cleaned DNA-free high-quality RNA was confirmed in a 1X bleach gel (Aranda et al., 2012).

### RNA-seq library preparation, illumina sequencing and data analysis

Strand-specific RNA-Seq libraries were constructed using the protocol described in Zhong et al. (2011) and sequenced on an Illumina HiSeq 2500 system using the single-end 100 bp mode. Raw RNA-Seq reads were processed to remove adaptor and low-quality sequences using Trimmomatic (Bolger et al., 2014) and to remove polyA/T tails using PRINSEQ++ (Cantu et al., 2019). RNA-Seq reads were then aligned to the ribosomal RNA database (Quast et al., 2013) using Bowtie (Langmead et al., 2009) and the mappable reads were discarded. The resulting high-quality cleaned reads were aligned to the tomato Heinz genome version SL4.0 (Hosmani et al., 2019) using HISAT2 (Kim et al., 2019). Following alignments, raw counts for each tomato gene were calculated and normalized to reads per kilobase of exon model per million mapped reads (RPKM). Raw counts were fed to the DESeq2 package (Love et al., 2014) to identify genes differentially expressed between the *ty-5* line and the S-line. Genes with adjusted p-values less than 0.05 and log2 fold changes above 1.5 or lower than -1.5 were identified as differentially expressed genes (**Supplementary Table S3**).

Differentially Expressed (DE) genes were used to perform Gene Ontology (GO) enrichment analysis utilizing the agriGO program (Du et al., 2010). The Tomato Functional Genomics Database (Fei et al., 2011) and the iTAK database (Zheng et al., 2016) were used to identify genes encoding plant transcription factors, and receptor-like kinases. Functional annotation of other genes of interest was performed using standalone BLAST (Altschul et al., 1990) by comparing homologs to genes of interest from *Arabidopsis* and *S. lycopersicum* in conjunction with utilizing annotated GO terms of tomato genes (Fernandez-Pozo et al., 2015).

A motif enrichment analysis was conducted using the MEME Suite (Bailey et al., 2009). For MEME analysis, promoter sequences (1000 bp upstream sequence of transcription start site) of differentially expressed defense-related genes were used. Motif enrichment was assessed with the SEA tool within the MEME Suite, which identified statistically enriched motifs in the provided sequences. Several of the enriched motifs showed significant similarity to known transcription factor binding motifs catalogued in the JASPAR CORE plants database (Sandelin et al., 2004).

### Validation of differentially expressed genes by qRT-PCR

Fourteen DEGs were randomly selected for validation by qRT-PCR. Primers were designed (**Supplementary Table S13**) and their specificity was confirmed by aligning the primer sequences to the tomato genome. cDNA was generated from 3 µg of the same tomato RNA preparations as those used for RNA-seq using the SuperScript III cDNA Synthesis System (ThermoFisher Scientific, USA). Twenty-five microliter PCR reactions consisted of 2 µL of diluted cDNA, 0.75 µL of each primer (10 µM), 12.2 µL of 2x Brilliant II SYBR Green Master Mix with low ROX (Agilent, USA), and 9.3 µL of nuclease-free water. PCR amplifications were performed in an Mx3005P Real-Time PCR System (Agilent, USA) using the following cycling conditions: 95 °C for 10 min., followed by 40 cycles of 95 °C for 30 seconds and 60 °C for 1 min with SYBR Green detection during the 60 °C step. The presence of a single amplicon in PCR reactions was confirmed by the presence of a single, uniform peak in dissociation curves conducted after amplification. Each of the selected genes was amplified from 3 biological replicates per treatment, with 3–4 technical replicates per biological replicate. Expression levels were normalized to the tomato actin gene (Solyc04g011500) using the ΔΔCt method and expressed in terms of log_2_(fold change) for comparison with the RNA-seq data. Significant differences in gene expression via qRT-PCR was determined using a one-tailed unpaired Student’s *t*-test (if data are normal and homoscedastic), Welch’s t-test (if heteroscedastic) or the Mann-Whitney Wilcox test (if not normally distributed). Statistical analysis was conducted in R (R Core Team, 2018).

## Results

### Transcriptome analysis on *ty-5* plants over a susceptible line upon TYLCV infection

**Figure 1:**
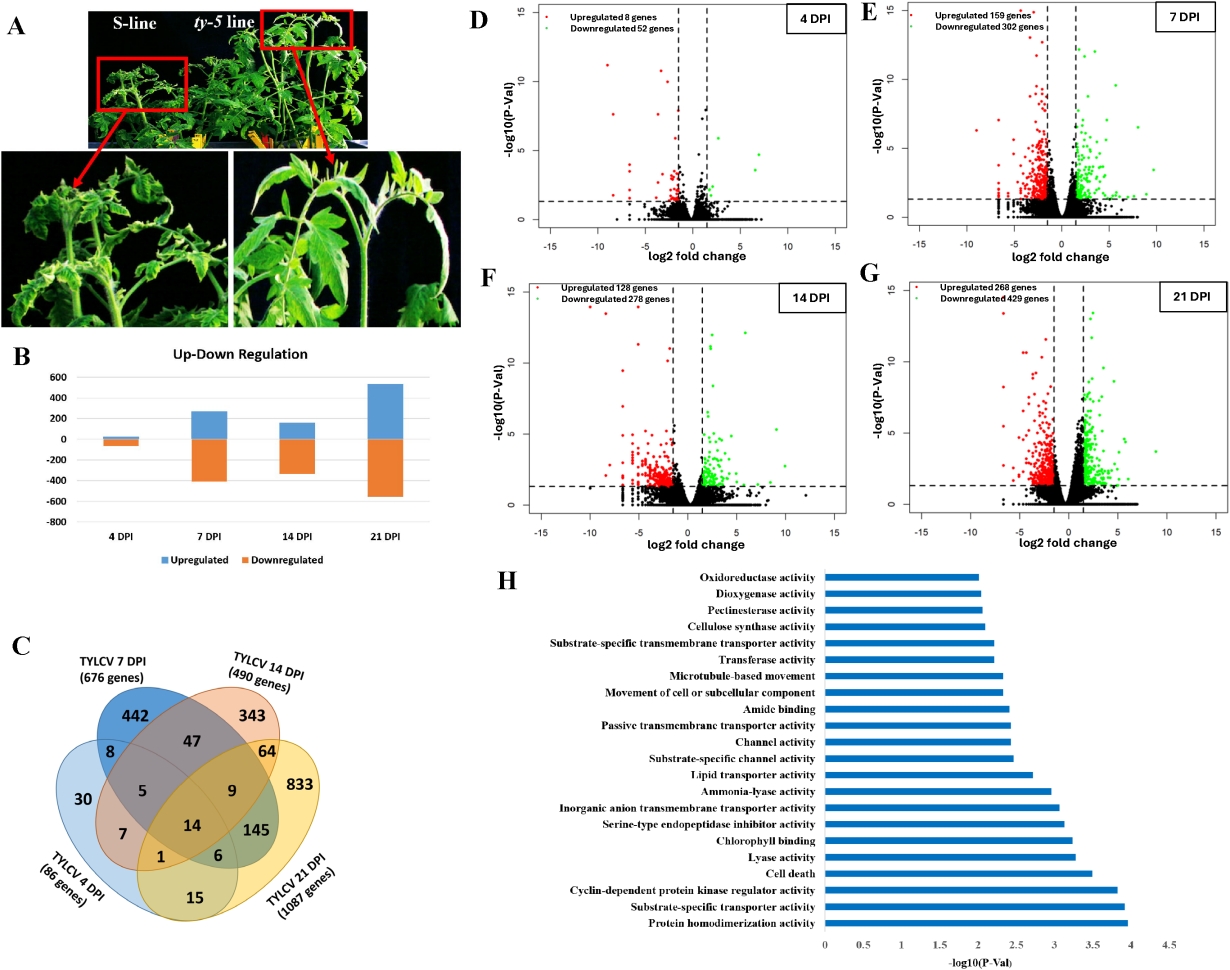
Differential gene expression between the resistant *ty-5* line and the susceptible S-line upon TYLCV infection. **(A)** Plants of the S-line (left) and the *ty-5* line (right) at 21 days post inoculation (dpi) with TYLCV. **(B)** Number of up- and down-regulated genes in the *ty-5* line compared to the S-line at 4, 7, 14, and 21 dpi with TYLCV. **(C)** Venn diagram showing the number of common, intersecting, and specific differentially expressed genes at 4, 7, 14, and 21 dpi. **(D-G)** Volcano plot showing 60, 461, 406, and 697 DEGs at 4 **(D)**, 7 **(E)**, 14 **(F)** and 21 **(G)** days post inoculation (dpi), respectively, with up-regulated shown in green and down-regulated in red. Black horizontal dotted lines indicate the p-value cutoff at 0.05 and black vertical dotted lines indicate log2(fold change) cutoffs at -1.5 and 1.5. **(H)** Gene Ontology (GO) enrichment analysis of the identified DEGs, showing enriched functional categories.

To obtain a global view on differential gene expression associated with *ty-5* mediated resistance to TYLCV, we conducted a comparative transcriptome analysis between a near-isogenic line carrying the *ty-5* resistance locus (hereafter referred to as the *ty-5* line) and its susceptible recurrent parent line (Fla. 8059, hereafter referred to as the S-line), using leaf tissue samples collected at four time points, 4-, 7-, 14-, and 21-days post inoculation (dpi) by the whitefly vector–spanning disease progression from inoculation to symptom expression. Typical disease symptoms, including leaf curling, yellowing, and stunting, were observed on the susceptible S-line plants around 14–21 dpi. During the same period, symptoms were very mild to almost absence from the TYLCV-inoculated *ty-5* plants (**Figure 1A**). From these leaf samples, a total of 24 RNA-Seq libraries were constructed and sequenced. Overall, an average of 11.68 million raw reads per library were obtained. After adapter trimming and removal of low-quality reads and rRNA sequences, an average of 10.04 million high quality clean reads were obtained per library, with 98.26% of those reads mapped to the tomato reference genome (version SL4.0) (**Supplementary Table S1**). We also conducted additional analysis by mapping of the RNA-seq data in each replicate to the TYLCV genome. The mapping results for the TYLCV genome are included in **Supplementary Table S1**. The TYLCV-related transcripts could be detected as early as 4 dpi. Some general trends were also observed: number of TYLCV-related transcripts increased over time (from 4 dpi to 21 dpi) and there were higher number of TYLCV-related transcripts in the susceptible control line (CT) over those of the *ty-5* line (TY5) at each sampling time point (4, 7, 14 and 21 dpi). However, since TYLCV is a DNA virus and this is an RNA-Seq dataset that was generated using a poly(A)-enriched library construction strategy, these results may not accurately reflect the true viral transcript abundance. Nevertheless, these results provided direct evidence to support an efficient transmission of TYLCV to the test plant by viruliferous whiteflies. Moreover, lower numbers of TYLCV transcripts were detected in the *ty-5* plants than those generated from the susceptible controls (**Supplementary Table S1**). High Pearson correlation coefficients among most biological replicates indicated strong reproducibility (**Supplementary Table S2**). However, three libraries with relatively lower correlation coefficients (TY5_TYCLV_7dpi_rep1, CT_TYCLV_14dpi_rep2, CT_TYLCV_21dpi_rep3) were excluded from downstream differential expression analysis. Comparative expression analysis identified a total of 1,394 differentially expressed genes (DEGs) between the *ty-5* line and S-line plants across all four timepoints (**Figures 1B**, **Supplementary Table S3**). Major groups of DEGs included 186 defense-related genes, 152 transcription factors, 68 protein kinases, 102 phytohormone-associated genes, 72 cell wall-associated genes, 26 histone methylation pathway genes, and 62 photosynthesis-related genes (**Supplementary Table S4**). These DEGs were further classified into 43 functional categories (**Supplementary Table S5**). At the pre-symptom expression phase at 4 dpi, only 60 DEGs were identified–eight up-regulated and 52 down-regulated (**Figures 1B, D**). As symptom expressions progressed, the number of DEGs increased substantially. At 7 dpi, there were 461 DEGs with 159 up-regulated and 302 down-regulated (**Figures 1B, E**). In addition, of the 406 DEGs identified at 14 dpi, 128 were up-regulated and 278 down-regulated (**Figures 1B, F**). The observed 14 dpi total DEGs are lower, suggesting a counter-response against pathogens. At 21 dpi, when the disease symptoms had fully appeared on the susceptible S-line plants, a total of 697 DEGs were identified, with 268 up-regulated and 429 down-regulated in the *ty-5* line (**Figures 1B, G**). This surge in the number of DEGs was correlated with leaf curling, yellowing and stunting symptom expression (**Figure 1A**). Genes affected by TYLCV infection were not constant from one time point to another between the two lines; a Venn diagram analysis revealed that only 14 genes were differentially expressed at all time points (**Figure 1C**). Volcano plots showing distributions of differentially expressed genes (DEGs) are illustrated in **Figures 1D–G**. Gene Ontology (GO) term enrichment analysis identified 22 significantly enriched functional categories, with protein homodimerization activity, substrate-specific transport activity, and cyclin-dependent protein kinase regulator activity being the top three categories (**Figure 1H**).

### Validating the expressions of differentially expressed genes

Differential expressions of 14 randomly selected DEGs were validated by quantitative reverse transcription polymerase chain reaction (qRT-PCR). All genes tested by qRT-PCR were in complete agreement with the expression patterns (upregulation or downregulation) observed in the RNA-seq datasets (**Table 1**). Likewise, the differential expressions observed via qRT-PCR were also statistically significant (p < 0.05) for 12 of the 14 DEGs (excluding Solyc04g079420 and Solyc04g005610), suggesting the validity of the RNA-seq datasets generated and transcriptome analysis methods used in this experiment.

**Table 1:** Comparative expression levels of selected differentially expressed genes between RNA-seq and qRT-PCR for validation analysis.

Gene ID	Annotation	Timepoint	RNA-seq		qRT-PCR	
			log2fold	Adj P-value	log2fold	P-value
Solyc12g087830	MADS box transcription factor	4 dpi	-3.32	1.73E-11	-3.52	1.08E-05
Solyc04g079420	Disease resistance protein (CC-NBS-LRR)	7 dpi	1.76	0.0000	1.05	0.2013
Solyc10g075070	Non-specific lipid-transfer protein	7 dpi	2.55	7.32E-04	0.90	0.0137
Solyc03g116100	MYB transcription factor	7 dpi	2.15	0.0305	0.29	0.0008
Solyc04g005610	NAC domain transcription factor	7 dpi	2.88	6.49E-04	0.87	0.1743
Solyc01g091230	Receptor like kinase, RLK	7 dpi	-2.00	0.0004	-2.98	3.01E-07
Solyc07g052790	NBS-LRR, resistance protein	14 dpi	-1.74	0.0000	-1.60	3.63E-05
Solyc10g047240	Annexin	14 dpi	-1.56	0.0000	-1.69	1.87E-07
Solyc05g009790	Transcription factor	14 dpi	2.75	0.0087	0.23	0.0846
Solyc04g007000	APETALA2/Ethylene Responsive Factor (AP2/ERF)	14 dpi	3.25	2.19E-05	1.70	0.0367
Solyc03g095770	WRKY transcription factor 6	21 dpi	1.56	0.0001	0.37	0.0135
Solyc02g089550	Protein NSP-interacting kinase	21 dpi	-2.00	0.0000	-1.45	6.40E-06
Solyc09g084470	Proteinase inhibitor I	21 dpi	3.29	0.0000	3.32	1.73E-08
Solyc05g052270	Serine/Threonine-protein kinase	21 dpi	-1.56	0.0085	-2.20	0.0002

### Defense-related genes are modulated in *ty-5* resistant lines

Among the differentially expressed genes, many are involved in defense-related responses, including those encoding nucleotide-binding site leucine-rich repeat (NBS-LRR) proteins, Avr9/Cf-9 rapidly elicited protein, members of the pathogenesis-related protein family, and glycine-/proline-rich proteins (**Supplementary Table S6**).

NBS-LRR proteins play a crucial role in effector-triggered immunity in plants through pathogen recognition and initiation of the hypersensitive response. In our dataset, nine NBS-LRR-related genes were differentially expressed (**Supplementary Table S6**). Notably, one of these NBS-LRR genes exhibited up to a 4-fold higher expression level in the *ty-5* line, indicating an enhanced effector-triggered immunity response. Additionally, several Avr9/Cf-9 genes were induced in the *ty-5* line. These genes encode rapidly elicited proteins upon fungal infection and are known to trigger a cascade of signal transduction events that activates the downstream plant defense mechanisms.

Pathogenesis-related (PR) genes are widely recognized as markers of plant immune responses, as they are typically induced upon pathogen infection to restrict disease progression. Notably, the gene encoding pathogenesis-related protein 1a was 5.83 (upregulated) at 7 dpi, but -6.64 (down-regulated) at both 14 and 21 dpi (**Supplementary Table S6**). This strongly suggests that the immune response in the *ty-5* resistant line may have been triggered by 7 dpi and maintained during later stages of infection. We discovered many other genes in the pathogenesis-related protein family that were differentially expressed in our dataset. For instance, pathogenesis-related thaumatin family gene was induced 2.88-fold at 14 dpi, implicating its key role in pathogen resistance. A gene encoding an aspartic protease inhibitor was induced 2.58-fold at 14 dpi and 2.21-fold at 21 dpi in the *ty-5* line (**Supplementary Table S6**). Interestingly, a cysteine/histidine-rich 1 gene, implicated in defense signaling pathways, was induced 3.38-fold at 21 dpi in the *ty-5* line (**Supplementary Table S6**). Peroxidases (PR-9) are also members of the pathogenesis-related protein subfamily. Among a total of eight differentially expressed peroxidase genes identified, all were activated in the *ty-5* line, with one upregulated up to 5.06-fold at 21 dpi (**Supplementary Table S6**). Proteinase inhibitors (PR-6), another PR subfamily, also appeared to play a key role in the *ty-5*-mediated resistance. Six PR-6 genes were induced in the *ty-5* resistant line, including three showing 4- to 5-fold increases compared to the S-line (**Supplementary Table S6**). Additionally, ten zinc finger protein genes were also upregulated. Zinc finger proteins act as transcription factors that regulate gene expression and mediate plant defense responses. Together, these findings indicate that a strong immune response is activated in the *ty-5* resistant plants and these PR genes may be directly involved in limiting the progression of viral disease.

Glycine-rich proteins are also known to play key roles in plant defense by inducing pathogenesis-related proteins. Three glycine-rich protein genes were found to be upregulated in the *ty-5* line. Additionally, a proline-rich protein gene was also induced in the *ty-5* line at 7 dpi. Upregulation of these defense-related genes further supports the hypothesis that resistant plants launch a robust immune response to TYLCV, leading to limited viral replication and symptom development. Moreover, the methylation pathway related-genes, including those encoding cytosine-5 DNA methyltransferase 1 and RNA-dependent RNA polymerase, were up-regulated in the *ty-5* line (**Supplementary Table S6**), implicating epigenetic regulation as an additional mechanism contributing to TYLCV resistance.

Motif enrichment analysis with MEME identified 95 significantly enriched motifs, each appearing a variable number of times within the promoter regions of defense-related genes. Based on statistical significance and motif frequency across promoter sequences, the three most enriched motifs were SACGTGGCA, GCCGCCGCCGCCCS, and ACACGTG (**Supplementary Figure S1**; **Supplementary Table S14**). These motifs showed strong matches to the GBF2, EREB, and ABF2 transcription factor binding motifs, respectively, as annotated in the JASPAR CORE plants database (Sandelin et al., 2004). Since our study is focused on plant resistance to viral pathogens, the second motif, EREB, also known as the GCC-box is of particular interest due to its well-established role in regulating defense-related plant gene expression. Our analysis revealed 75 out of 128 defense-related genes contain at least one or more GCC-box motifs in their promoter sequences (**Supplementary Table S14**). Notably, two pathogenesis-related (PR) genes, Solyc08g080660 and Solyc02g031920, harbor this motif within their cis-regulatory promoter sequences, highlighting a potential regulatory link between GCC-box–mediated transcriptional control and antiviral defense responses.

### Many transcription factor genes responded to the TYLCV infection

Transcription factors (TFs) play a central role in regulating gene expression and are often modulated by plant hormones such as jasmonic acid (JA), salicylic acid (SA), and ethylene. In this study, a total of 112 annotated transcription factor genes were differentially expressed, with 52 upregulated in the TYLCV resistant *ty-5* line, 49 downregulated (**Supplementary Table S7**; **Supplementary Figure S2**) and the remaining 11 up- and down-regulated at different time points. Five MADS-box TFs were differentially expressed, including one that was induced more than five-fold at 7 dpi. Among six NAC domain TFs with differential expression, three were upregulated in the *ty-5* line, with one showing a 3.66-fold increase. Among 23 differentially expressed MYB family TFs, nine were upregulated in the *ty-5* line, including two with more than 3-fold induction. A prior study has demonstrated that suppression of MYB TFs compromises *N* gene-mediated resistance against tobacco mosaic virus (an RNA virus) (Zhu et al., 2022). However, the role of MYB TFs in resistance to single-stranded DNA (ssDNA) viruses, such as begomoviruses, remains unclear. In addition, seven WRKY TFs showed altered expression, with five upregulated in the *ty-5* line and two others were downregulated. WRKY TFs are key players in disease resistance and plant immunity.

### More protein kinases were down-regulated in the *ty-5* line

By phosphorylating target proteins to switch them from inactive to active states, protein kinases often function within signaling cascades. Amon DEGs identified in this study, 49 protein kinase genes showed altered expression at various time points (**Supplementary Table S8**). Of these, seven were upregulated in the *ty-5* plants following TYLCV infection, 40 were downregulated, and the remaining two exhibited up- and down-regulation at different time points. The upregulation of protein kinases in the S-line may reflect their multiple roles, including activation of viral proteins, host susceptibility factors, or downstream signaling components to promote disease. Notably, a substantial number of protein kinase genes (32) were upregulated in the S-line at 21 dpi, coinciding with the appearance of disease symptoms at 21 dpi. Many of these genes encode LRR receptor-like kinase family proteins and receptor-like protein kinases. Interestingly, one G-type lectin S-receptor-like serine/threonine-protein kinase gene was induced nearly four-fold in the *ty-5* line, suggesting a potential role in defense signaling. However, the exact roles of these kinases remain unclear, as they might activate or trigger defense-related genes and downstream signaling compounds, which could also activate susceptibility factors.

### Phytohormones are signal molecules regulating plant growth and development

A total of 94 DEGs related to phytohormones were identified in our dataset (**Supplementary Table S9**). Among them, 23 were upregulated up to 4.3-fold in the *ty-5* resistant line. We also identified nine differentially expressed glutaredoxin genes, with five upregulated in the *ty-5* line (**Supplementary Table S9**). Previous studies have shown that expression of a glutaredoxin in *Nicotiana benthamiana* plants suppresses pepper mild mottle virus-Italian (PMMoV-I) accumulation and correlates with induction of SA-regulated PR-proteins (Hakmaoui et al., 2012). Consistently, in addition to the upregulation of glutaredoxin, we also observed enhanced expression of several PR genes in the resistant line (**Supplementary Table S6**).

### Cell-to-cell transport of macromolecules

Cell wall-associated proteins play a key role in cell-to-cell transport of macromolecules, including nucleic acids, gene silencing signals, proteins from host and pathogen, the siRNA/microRNA-associated RISC complex, plant viruses, and plastids. In our dataset, 64 cell wall-related genes were differentially expressed at different time points (**Supplementary Table S10**). Eleven of these genes were upregulated in the *ty-5* plants after TYLCV infection and the rest were downregulated (**Supplementary Table S10**). Notably, five germin-like protein genes were highly induced (ranging from 5- to 7-fold) in the S-line after TYLCV infection over the course of infection (4, 7, 14, and 21 dpi) (**Supplementary Table S10**), which may facilitate viral infection in S-line.

### Photosynthesis genes and virus resistance

Chloroplast-related genes could be involved in plant-virus interactions. A total of 35 DEGs related to photosynthesis were identified in our dataset (**Supplementary Table S11**). Most of these genes (19) were upregulated (up to 4.3-fold) in the *ty-5* resistant line. It was reported that some photosynthesis-related genes are involved in plant resistance to viruses, such as soybean mosaic virus (Bwalya et al., 2022).

### Histone remodeling and RNA-directed DNA methylation

Histone proteins are essential for DNA packaging into nucleosomes and chromatin structure maintenance. Each nucleosome is comprised of a core of histones: H1/H5, H2A, H2B, H3, and H4. Histone methyltransferases can modify histone proteins at certain sites through methylation, thus regulating gene expression by altering chromatin accessibility. In this study, all five histones (H1, H3, H2A, H2B, and H4), as well as chromatin remodeling-related genes such as those encoding a histidine phosphotransferase protein, a histone-lysine N-methyltransferase, and a high mobility group protein, were upregulated in the S-line. Notably, 22 of these genes were upregulated at 7 dpi and four were upregulated at 21 dpi (**Supplementary Table S12**). Upregulation of these components in the S-line may be the result of the virus establishing its replication complex in the nucleus, although the exact role they play in host susceptibility remains elucidated.

DNA methylation is a key epigenetic mechanism involving the addition of methyl groups to cytosine residues in DNA, a process catalyzed by DNA methyltransferases, including DNA (cytosine 5) methyltransferase 3. RNA-directed DNA methylation involves *de novo* methylation of cytosine residues of DNA in the chromatin structure that share complementary sequences with small RNA, forming transcriptionally silent heterochromatin. In this study, cytosine-5 DNA methyltransferase 1 was induced ~2-fold at 7 and 14 dpi in the *ty-5* line (**Supplementary Table S7**). This enhanced expression may contribute to RdDM-mediated silencing of the TYLCV genome in ty-5 plants, thus restricting virus replication and conferring resistance.

RNA silencing is a major antiviral defense mechanism in both plants and animals. During viral infection, double-stranded RNA (dsRNA) molecules are generated either as replicative intermediates or through the formation of secondary dsRNA structures, which are recognized by specific Dicer-like proteins and cleaved into 21–24 nt siRNAs. In plants, 22-nt siRNAs can serve as primers for the host-encoded RNA-dependent RNA polymerase (RDRP)-mediated conversion of long dsRNAs into secondary siRNAs. In this way, silencing signal not only is being amplified but is also spread along the entire RNA target sequence as trans-acting siRNA (tasiRNAs). The tomato genome encodes seven DICER-like endonucleases (DCLs), 15 Argonautes (AGOs), and six RNA-dependent RNA polymerase (RDRs) genes. In this study, we identified an RDR of the RDR_ϒ_ type, harboring an atypical DFDGD motif in the catalytic domain, that was upregulated 2.0 to 3.8-fold at 4, 7, and 14 dpi in the *ty-5* line, suggesting a defense response in *ty-5* plants potentially through DNA methylation of the TYLCV genome (**Supplementary Table S7**). Considering that Ty-1/Ty-3 resistance genes against TYLCV are RDRPs (Verlaan et al., 2013), it is interesting that an RNA–dependent RNA polymerase (RDR) gene was upregulated in the *ty-5* line.

### Functional characterization of APETALA2/ethylene responsive factor

**Figure 2:**
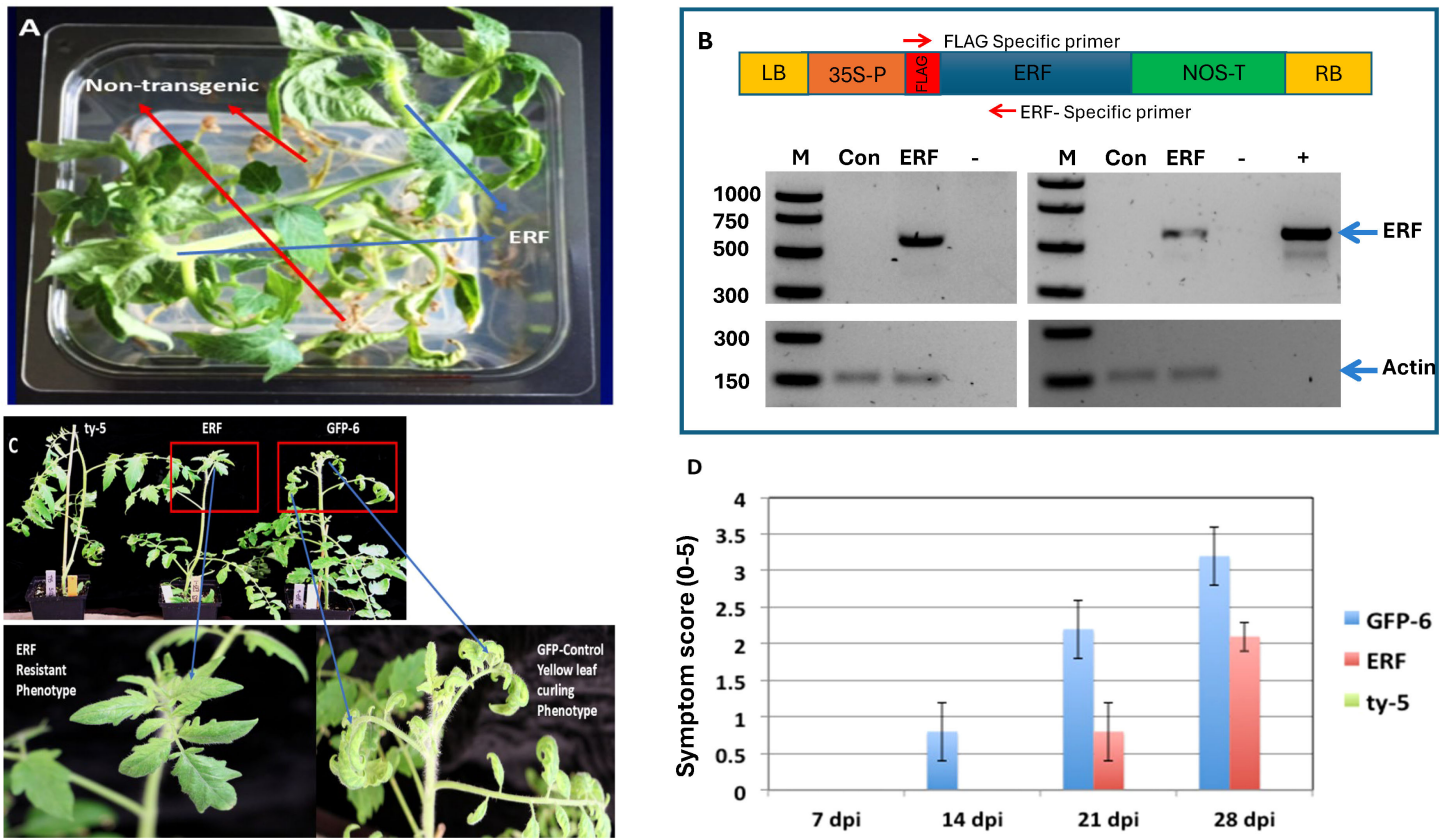
Functional characterization of the APETALA2/Ethylene Responsive Factor (AP2/ERF TF) gene for its contribution to resistance against TYLCV. **(A)** Transgenic plant lines were regenerated on selective media containing phosphinothricin, where non-transformed plants could not survive. **(B)** The AP2/ERF gene was cloned and inserted into the binary vector (pEG101) between a 35S promoter and a NOS terminator with an N-terminus FLAG tag (upper panel). PCR analysis (left) confirmed the presence of an AP2/ERF transgene and RT-PCR (right) showed the expression of the transgene (AP2/ERF) alongside positive (+) and negative (−) controls as well as GFP transgenic plants. Actin serves as internal control in both cases. **(C)** Evaluation of transgenic lines in response to TYLCV infection via viruliferous whiteflies: AP2/ERF over-expressing line showed resistance to TYLCV with no visible symptoms, whereas control plants (GFP-Moneymaker) displayed leaf curling and disease upon TYLCV infection. **(D)** Disease severity symptom scores on transgenic line AP2/ERF (ERF), transgenic GFP6 as vector control (GFP-6), and *ty-5* resistant line (ty-5) upon whitefly feeding inoculation of TYLCV were visually assessed for their symptom scores at 7, 14, 21, and 28 dpi, respectively. Each plant was rated on a scale of 0–5 symptom severity scores as described (Lapidot et al., 2006). Data shown represents average symptom scores from three experiments (five plants per experiment), and bars indicate standard errors. There were no visual symptoms observed on test plants in the *ty-5* line.

Based on the transcriptome analysis, several defense-related genes were induced in the *ty-5* line, including a member of the AP2/ERF TF family, which is known to promote the expression of pathogenesis-related family protein genes (Su et al., 2025). An earlier study also identified 22 tomato AP2/ERF TFs responding to TYLCV infection (Huang et al., 2016). To better understand the role of this gene in the *ty-5*-mediated resistance, we over-expressed this gene in the susceptible tomato cultivar ‘Moneymaker’ (**Figure 2B**). Eight independent phosphinothricin resistance transgenic lines were generated and used for evaluation for TYLCV resistance through whitefly transmission (**Figure 2A**).

Disease progression and symptom development were monitored in transgenic plants overexpressing AP2/ERF and in control plants expressing green fluorescent protein (GFP), following inoculation of the plants by 3,000 TYLCV-viruliferous whiteflies in a greenhouse. Typical TYLCV disease symptoms were observed as early as 14–21 dpi in the control plants (**Figure 2C**). In contrast, AP2/ERF-overexpressing plants showed no visible symptoms at 14 dpi and exhibited milder symptoms at 21 and 28 dpi (**Figure 2C**). Moreover, fewer inoculated AP2/ERF-overexpressing plants became infected with TYLCV than the GFP transgenic controls (**Figure 2C**). TYLCV was not detectable in any of the AP2/ERF-overexpressing plants until 21 dpi, whereas it was already detected in some GFP-expressing plants at 14 dpi (**Figure 2D**). The appearance of symptoms at a later stage might be a reflection of resistance associated with multiple genes.

## Discussion

We present a comprehensive global transcriptome analysis of tomato response to TYLCV in the context of the recessive *ty-5* resistance gene via natural whitefly-mediated inoculation. We identified a total of 1,394 differentially expressed genes, spanning a wide range of biological processes, including many defense-related genes and those involved in gene regulation and silencing, highlighting the multifaceted nature of the resistance response conferred by *ty-5*. Although most of the transcriptome studies are conducted typically through a comparison of differential gene expressions between treatment and its mock control. In the present study, we chose to use a near isogenic line (NIL) and its recurrent parent upon TYLCV infection for transcriptome analysis. A NIL is genetically identical to the recurrent parent except in a small genomic region with the unique trait of interest, as stated in the materials and methods, these two lines theoretically share 96.875%. Using NIL for transcriptome analysis is a powerful strategy to identify the candidate genes, which may allow us to uncover the molecular mechanism underlying a specific trait of interest (TYLCV resistance by *ty-5*). By comparing the gene expression profiles of the NIL line and its recurrent parent, researchers can effectively filter out the “genetic noise” to concentrate on transcriptional changes that are likely associated with the trait of interest, such as disease resistance (Escalante et al., 2023; Habib et al., 2018) or drought tolerance (Nouraei et al., 2022). A similar strategy was used in one of our earlier studies to report transcriptome of tomato spotted wilt virus (TSWV)-inoculated tomato plants between a NIL and the recurrent parental line for their differential gene expression at different time points post virus inoculation (Padmanabhan et al., 2019). Therefore, the transcriptome analysis in the present study to compare differential gene expressions between a NIL line and its recurrent parent upon TYLCV infection at four different time intervals would allow us to identify genes that could be most likely associated with the *ty-5* resistance.

By conducting transcriptome analysis of differential gene expression at multiple time points post virus-inoculation, it may be possible to identify the dynamic, temporal and spatial mechanisms of host-virus interactions. The time course analysis can distinguish between early responding genes such as those for antiviral factors and late-stage responses including tissue damage or recovery. Therefore, it is possible to observe differential expressions of certain genes from one time point to another, particularly between in the early stages over late-stage post virus inoculation.

Our data indicates that many defense-related genes were induced in the resistant *ty-5* line, particularly NBS-LRR and pathogenesis-related genes. NBS-LRR proteins play a central role in effector-triggered immunity in plants and are known to bind to pathogen effectors and trigger the hypersensitive response, which effectively restricts the systemic spread of biotrophic pathogens such as viruses (DeYoung and Innes, 2006; McHale et al., 2006; Moffett et al., 2002). In fact, the resistance gene *Ty-2* which encodes an NBS-LRR protein and interacts with the Rep/C1 protein confers the species-specific resistance to TYLCV (Shen et al., 2020).

**Figure 3:**
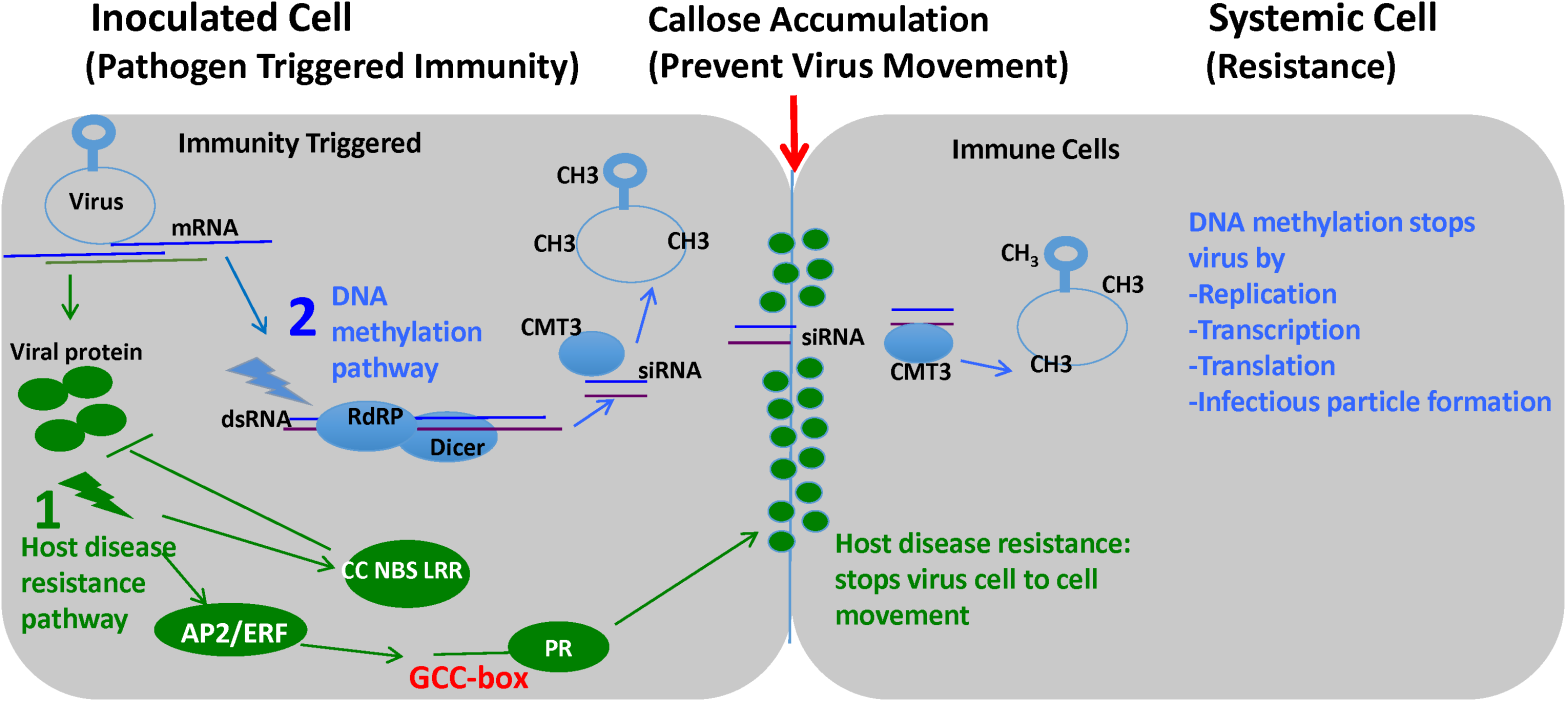
A schematic model illustrating TYLCV disease resistance in the *ty-5* line. The *ty-5* line with resistance to TYLCV likely involved two pathways in tomato: 1. host resistance (green) and 2. DNA methylation pathways (blue), which activated cooperatively to restrict virus spread cell-to-cell and systemic movement. Activation of the host disease resistance pathway occurs through CC NBS LRR recognition of viral proteins and expression of pathogenesis-related (PR) genes (with GCC-box) through transcription factors such as APETALA2/Ethylene Responsive Factor (AP2/ERF TF) which, in turn, leads to enhanced callose deposition along the cell wall, limiting virus movement. RNA-mediated DNA methylation starts with the generation of siRNAs by the activity of Dicer and secondary siRNAs through amplification by RNA-dependent RNA polymerases (RDRPs). siRNAs serve as a template for methylation (CH3) of the viral genome via Cytosine-5 DNA methyltransferase 1. This methylation prevents virus replication and transcription, thus inhibiting virus protein expression and eventual infectious particle formation.

Pathogenesis-related (PR) genes are widely recognized as markers for plant disease resistance, as they are typically induced upon pathogen infection to restrict disease spread (Ali et al., 2018). The observed induction of PR genes (**Supplementary Table S6**) might play an important role in the immunity during TYLCV infection by promoting callose deposition in the cell wall (Montasser et al., 2012) to restrict viral movement and possibly activating systemic acquired resistance (**Figure 3**). Glycine/proline rich proteins play a key role in disease resistance in plants by inducing pathogenesis-related proteins (Czolpinska and Rurek, 2018). In plant-virus interactions, peroxidase (PR-9) activity has been associated with infection by a variety of viruses, including TMV, white clover mosaic potexvirus, cucumber mosaic virus, zucchini yellow mosaic virus, and pepper yellow mosaic virus (Clarke et al., 2002; Radwan et al., 2007; Goncalves et al., 2013). Our study adds TYLCV to this list. We also noted that members of the glycine/proline-rich protein families were upregulated in the *ty-5* line. Prior studies have demonstrated that glycine-rich proteins play a role in lignin biosynthesis and/or deposition (Chen et al., 2007), and more importantly, promote callose deposition in the cell wall to block virus systemic spread (Ueki and Citovsky, 2002; 2005). Therefore, the increased expression of these glycine-rich protein genes in the *ty-5* line might serve as a positive regulator of PR gene-mediated callose deposition, enhancing disease resistance in tomato plants.

Numerous protease inhibitors were also upregulated in the ty-5 plants, particularly at the late infection stage (21 dpi). Previous studies have shown that RNAi-mediated suppression of protease inhibitor genes can compromise resistance to potyviruses, such as papaya ringspot virus in watermelon (Lin et al., 2015). A novel strategy in engineering a pathogen-derived protease cleavage site in frame with an NLR gene confers a broad-spectrum resistance to multiple potyviruses (Wang et al., 2025). In potyviruses, viral genomic RNA is translated into a polyprotein, which is cleaved by the viral-encoded proteases into active functional products that enable virus infection. Overexpression of host protease inhibitors can block viral proteases to properly process the viral polyprotein, thus suppressing virus replication. However, no proteases are encoded in the ssDNA TYLCV genome. Hence, the observed strong upregulation of eight protease inhibitors in the *ty-5* resistant line and their roles in resistance against TYLCV requires further investigation.

Among the 23 MYB family TFs with altered expression in the *ty-5* line, nine were upregulated primarily at late infection stages (14 dpi and 21 dpi), with one showing more than 3-fold induction. Suppression of the MYB TFs compromises *N* gene-mediated resistance against TMV, an RNA virus (Liu et al., 2004). Similarly, of the seven WRKY TFs with altered expression, five were upregulated in the *ty-5* line at 14 or 21 dpi. WRKY TFs are key players of disease resistance and plant immunity (Bakshi and Oelmüller, 2014). Zinc finger antiviral proteins specifically inhibit the replication of viruses in animals (Guo et al., 2002). In this study, eight zinc finger protein genes showed differential expression, including a zinc finger CCCH domain protein gene altered at 14 dpi and 21 dpi. Zhang et al. (2024) demonstrated that topical application of gibberellic acid (GA3) on tomato plants, not only induced the expression of WRKYs, NACs, MYBs, P450 and ERFs, but also resulted in tomato resistance to TYLCV infection.

Protein kinases play critical roles in almost all biological processes and several disease resistance genes encode kinase motifs involved in triggering immunity against various pathogens, including begomoviruses (Shen et al., 2014). Protein kinases are a large gene family in plants that play an important role in disease resistance by phosphorylating other proteins from an inactive to an active stage and are often involved in signaling cascades (Tang et al., 2017). In TYLCV, protein kinases can have a dual role in plants response to the virus infection, some enhance the resistance, while others evade the defense system resulting in susceptibility. It has been demonstrated that the C4 protein of TYLCV can broadly interact with plant receptor-like kinases (RLKs), suggesting the C4 is a potential manipulator of the RLK-mediated signaling pathway (Garnelo Gómez et al., 2019). In another example, SlMAP3 regulates the salicylic acid and jasmonic acid signaling pathways in tomato plants, resulting with an enhanced tolerance to TYLCV (Li et al., 2017). In the present study, three Serine/Threonine-protein kinases were upregulated in the *ty-5* line. Further investigation is needed to characterize their functions associated with the *ty5* resistance.

Cell wall associated proteins play a key role in cell-to-cell transport of macromolecules, including nucleic acids, gene silencing markers, proteins from host and pathogen, the siRNA/microRNA associated RISC complex, plant viruses, and plastids (Houston et al., 2016).

Several proteins involved in callose deposition in the cell wall, such as callose synthase, pattern recognition receptors, and kinases, are involved in protein phosphorylation. Calcium ions play a vital role in promoting callose production in plants and the callose deposition on plasmodesmata in cell walls could increase disease resistance by restricting the virus movement from cell to cell (Li et al., 2023).

RNA-directed DNA methylation genes confer TYLCV resistance in the *ty-5* Line. RNA-directed DNA methylation has been observed in plants and is mediated by siRNA. Briefly, when siRNA forms a complex with complementary DNA sequences, nearly all cytosine residues in that region of DNA are methylated. However, this process involves the cooperation of many components, such as the 24 nt small RNA itself and other proteins such as DCL3, RDR2, AGO4, PolIV and PolV (Pikaard, 2013). In our study, we observed two RNA directed DNA methylation pathway genes (Cytosine-5 DNA methyltransferase 1 and RNA-dependent RNA polymerase) that were upregulated in the *ty-5* line. Among these was an RDRP, an enzyme that converts single stranded RNA into double-stranded RNA, which is the precursor for siRNA generation. Once the RNA is made double-stranded, it is recognized and cleaved by DCL3, and then a single piece is loaded into the AGO4 protein. The siRNA then guides the complex to a complementary strand of DNA, where methyl groups are added to the cytosine residues by DNA methyltransferases such as DNA (cytosine-5)-methyltransferase 3 (Jin and Robertson, 2013). Interestingly, in our dataset we also identified a DNA cytosine-5 DNA methyltransferase 1 that was upregulated in the *ty-5* line. These data suggest that the RDRP, along with the DNA (cytosine-5)-methyltransferase work together to enhance methylation of the TYLCV DNA genome, resulting in shutdown of viral DNA replication/transcription in the *ty-5* line (**Figure 3**). A similar mechanism was found to occur in the context of *Ty-1/Ty-3*, which is also an RDRP (Verlaan et al., 2013); in this case, *Ty-1/Ty-3* contributes to hypermethylation of the viral genome, specifically the V1 promoter region, thereby causing transcriptional silencing of the viral genome and resulting in resistance (Butterbach et al., 2014).

Notably absent from the list of DEGs is *Pelo*, the *ty-5* resistance gene itself. However, the fact that *Pelo* was not determined to be differentially expressed is not surprising because the difference between susceptible and resistant alleles of this gene is at the protein level and not the transcript level. When Lapidot et al. (2015) first cloned the *Pelo* gene from the *ty-5* locus, they noticed a single base pair difference between resistant and susceptible genotypes, resulting in a non-synonymous amino acid mutation of valine to glycine in the resulting protein. Hence, this is a difference in protein composition rather than relative expression, and we therefore would not expect to observe *Pelo* as differentially expressed gene in our dataset. The *ty-5* resistance gene, *Pelo*, is a part of a translation quality control process known as no-go decay that feeds directly into the RNAi pathway and possibly the RNA-directed DNA methylation pathway as well (Li et al., 2025). When a ribosome stalls on an aberrant transcript, *Pelo* “rescues” the ribosome, releasing the problematic transcript, which is then cleaved and serves as substrate for the generation of siRNAs by RDRPs (Szadeczky-Kardoss et al., 2018). This connection between the *ty-5* resistance gene *Pelo* and the RDRP *Ty-1*/*Ty-3* resistance gene may explain why genetic interaction has previously been observed between these resistance genes (Vidavski et al., 2008).

The AP2/ERFs binds specifically to the GCC box in the promoters of ethylene-inducible pathogenesis-related (PR) genes. The identification of motifs in the promoter regions in differentially expressed defense-related genes revealed many of them (75 of 128) with GCC box (core sequence GCCGCC) (**Supplementary Table S14**). The AP2/ERF factor ORA59 binds to two GCC boxes in the *Plant Defensin1.2* (*PDF1.2*) promotor to activate plant defense gene expression through the JA and ET signaling pathways in *Arabidopsis* (Zarei et al., 2011). In plant disease resistance, AP2/ERFs act on downstream of mitogen activated protein kinase (MAPK) cascades to regulate the expression of genes associated with the hormonal signaling pathways, secondary metabolites, and physical barriers (Ma et al., 2024). The selected AP2/ERF gene used in the present study was specifically induced once at 14 dpi. At this stage, the susceptible plants would begin to induce disease symptom expression. Other AP2/ERFs were more dynamic in their expression patterns, although some of them may also contribute to the *ty-5* resistance, which could be used in a future study. In a previous study, Huang et al. (2016) elegantly demonstrated the binding of several AP2/ERFs to the GCC box using Yeast one-hybrid, although which Ty gene in their TYLCV resistant tomato cultivars used in that study was not specified. Through over-expression in transgenic tomato plants to one of the AP2-ERFs, the present study offered the first evidence to document contribution of AP2/ERFs in the *ty-5* resistance to TYLCV, in addition to Pelota. In addition, we identified two differentially expressed PR genes (Solyc08g080660 and Solyc02g031920) with the GCC box in their cis-regulatory promoter sequences (**Supplementary Table S14**), which may contribute to the *ty-5* resistance to TYLCV (**Figure 3**). On the other hand, the *ty-5* (Pelota) with a single nucleotide mutation to the *pelo* gene affects an RNA surveillance factor by reducing its translational efficiency that inhibits directly the viral replication (Lapidot et al., 2015). Further study is needed to uncover how these pathways work cooperatively to confer the recessive resistance to TYLCV by *ty-5*.

Therefore, we propose a two-way mechanism for the *ty-5* resistance: one involving the initiation of defense-related gene expression and the other involving RNA-directed DNA methylation. To test the first hypothesis, we investigated the role of the AP2/ERF protein in TYLCV resistance. To investigate its functional role in the defense response of TYLCV, we over-expressed AP2/ERF in a susceptible tomato line. Interestingly, after TYLCV inoculation, the AP2/ERF over-expressing transgenic plants showed only moderate resistance to TYLCV infection in comparison to the control plants, suggesting the need for Pelota to achieve high resistance. These data support our hypothesis that increased expression of the AP2/ERF transcription factor promotes resistance to TYLCV, possibly through the induction of the cluster of PR-proteins, which restrict virus cell-to-cell movement through callose deposition in the cell wall (**Figure 3**). This response likely operates in parallel to the RNA-directed DNA methylation pathway which shuts down virus replication, working together to generate robust resistance to TYLCV. Further study will be needed to confirm the RNA-directed DNA methylation as well as the potential interplay between them. In addition to its high resistance to TYLCV, the *ty-5* gene has been demonstrated to offer a broad-spectrum resistance to several other Geminiviruses (Ren et al., 2022) indicating the importance of incorporating the *ty-5* gene in tomato breeding.

## Data Availability

RNA-seq datasets were submitted to NCBI SRA database with the accession No. PRJNA1389819.

## References

[B1] Ali S. Ganai B. A. Kamili A. N. Bhat A. A. Mir Z. A. Bhat J. A. . (2018). Pathogenesis-related proteins and peptides as promising tools for engineering plants with multiple stress tolerance. Microbiol. Res. 212-213, 29–37. doi: 10.1016/j.micres.2018.04.008. PMID: 29853166

[B2] Altschul S. F. Gish W. Miller W. Myers E. W. Lipman D. J. (1990). Basic local alignment search tool. J. Mol. Biol. 215, 403–410. doi: 10.1016/s0022-2836(05)80360-2. PMID: 2231712

[B3] Anbinder I. Reuveni M. Azari R. Paran I. Nahon S. Shlomo H. . (2009). Molecular dissection of Tomato leaf curl virus resistance in tomato line TY172 derived from Solanum Peruvianum. Theor. Appl. Genet. 119, 519–530. doi: 10.1007/s00122-009-1060-z. PMID: 19455299

[B4] Aranda P. S. LaJoie D. M. Jorcyk C. L. (2012). Bleach gel: a simple agarose gel for analyzing RNA quality. Electrophoresis 33, 366–369. doi: 10.1002/elps.201100335. PMID: 22222980 PMC3699176

[B5] Bailey T. L. Bodén M. Buske F. A. Frith M. Grant C. E. Clementi L. . (2009). MEME Suite: tools for motif discovery and searching. Nucleic Acids Res. 37, W202–W208. doi: 10.1093/nar/gkp335. PMID: 19458158 PMC2703892

[B6] Bakshi M. Oelmüller R. (2014). WRKY transcription factors: Jack of many trades in plants. Plant Signal. Behav. 9, e27700. doi: 10.4161/psb.27700. PMID: 24492469 PMC4091213

[B7] Bolger A. M. Lohse M. Usadel B. (2014). Trimmomatic: a flexible trimmer for Illumina sequence data. Bioinformatics 30, 2114–2120. doi: 10.1093/bioinformatics/btu170. PMID: 24695404 PMC4103590

[B8] Boykin L. M. De Barro P. J. (2014). A practical guide to identifying members of the Bemisia tabaci species complex: and other morphologically identical species. Front. Ecol. Evol. 2. doi: 10.3389/fevo.2014.00045. PMID: 41859668

[B9] Butterbach P. Verlaan M. G. Dullemans A. Lohuis D. Visser R. G. Bai Y. . (2014). Tomato yellow leaf curl virus resistance by Ty-1 involves increased cytosine methylation of viral genomes and is compromised by cucumber mosaic virus infection. Proc. Natl. Acad. Sci. U.S.A. 111, 12942–12947. doi: 10.1073/pnas.1400894111. PMID: 25136118 PMC4156758

[B10] Bwalya J. Alazem M. Kim K. H. (2022). Photosynthesis-related genes induce resistance against soybean mosaic virus: Evidence for involvement of the RNA silencing pathway. Mol. Plant Pathol. 23, 543–560. doi: 10.1111/mpp.13177. PMID: 34962034 PMC8916206

[B11] Cantu V. A. Sadural J. Edwards R. (2019). PRINSEQ++, a multi-threaded tool for fast and efficient quality control and preprocessing of sequencing datasets. PeerJ Prepr. 7, e27553v1. doi: 10.7287/peerj.preprints.27553v1

[B12] Caro M. Verlaan M. G. Julián O. Finkers R. Wolters A. M. Hutton S. F. . (2015). Assessing the genetic variation of Ty-1 and Ty-3 alleles conferring resistance to tomato yellow leaf curl virus in a broad tomato germplasm. Mol. Breed. 35, 132. doi: 10.1007/s11032-015-0329-y. PMID: 26028987 PMC4442973

[B13] Chen T. Lv Y. Zhao T. Li N. Yang Y. Yu W. . (2013). Comparative transcriptome profiling of a resistant vs. susceptible tomato (Solanum lycopersicum) cultivar in response to infection by tomato yellow leaf curl virus. PloS One 8, e80816. doi: 10.1371/journal.pone.0080816. PMID: 24260487 PMC3832472

[B14] Chen A. P. Zhong N. Q. Qu Z. L. Wang F. Liu N. Xia G. X. (2007). Root and vascular tissue-specific expression of glycine-rich protein AtGRP9 and its interaction with AtCAD5, a cinnamyl alcohol dehydrogenase, in Arabidopsis thaliana. J. Plant Res. 20, 337–343. doi: 10.1007/s10265-006-0058-8. PMID: 17287892

[B15] Clarke S. F. Guy P. L. Burritt D. J. Jameson P. E. (2002). Changes in the activities of antioxidant enzymes in response to virus infection and hormone treatment. Physiol. Plant 114, 157–164. doi: 10.1034/j.1399-3054.2002.1140201.x. PMID: 11903962

[B16] Cohen S. Nitzany F. E. Harpaz I. (1963). Tests for the control of tomato top yellowing virus. Hassadeh (in Hebrew) 43, 576–578.

[B17] Czolpinska M. Rurek M. (2018). Plant glycine-rich proteins in stress response: an emerging, still prospective story. Front. Plant Sci. 8. doi: 10.3389/fpls.2018.00302. PMID: 29568308 PMC5852109

[B18] DeYoung B. J. Innes R. W. (2006). Plant NBS-LRR proteins in pathogen sensing and host defense. Nat. Immunol. 7, 1243–1249. doi: 10.1038/ni1410. PMID: 17110940 PMC1973153

[B19] Du Z. Zhou X. Ling Y. Zhang Z. Su Z. (2010). agriGO: a GO analysis toolkit for the agricultural community. Nucleic Acids Res. 38, W64–W70. doi: 10.1093/nar/gkq310. PMID: 20435677 PMC2896167

[B20] El-Sappah A. H. Qi S. Soaud A. S. Huang Q. Saleh A. M. Abourehab A. S. M. . (2022). Natural resistance of tomato plants to Tomato yellow leaf curl virus. Front. Plant Sci. 13, 1081549. doi: 10.3389/fpls.2022.1081549, PMID: 36600922 PMC9807178

[B21] Escalante C. Sela N. Valverde R. A. (2023). Transcriptome analysis of two near-isogenic lines of bell pepper (Capsicum annuum) infected with bell pepper endornavirus and pepper mild mottle virus. Front. Genet. 14. doi: 10.3389/fgene.2023.1182578. PMID: 37124621 PMC10133535

[B22] Fei Z. Joung J. G. Tang X. Zheng Y. Huang M. Lee J. M. . (2011). Tomato Functional Genomics Database: a comprehensive resource and analysis package for tomato functional genomics. Nucleic Acids Res. 39, D1156–D1163. doi: 10.1093/nar/gkq991. PMID: 20965973 PMC3013811

[B23] Fernandez-Pozo N. Menda N. Edwards J. D. Saha S. Tecle I. Y. Strickler S. R. . (2015). The Sol Genomics Network (SGN) from genotype to phenotype to breeding. Nucleic Acids Res. 43, D1036–D1041. doi: 10.1093/nar/gku1195. PMID: 25428362 PMC4383978

[B24] Garnelo Gómez B. Zhang D. Rosas-Díaz T. Wei Y. Macho A. P. Lozano-Durán R. (2019). The C4 protein from tomato yellow leaf curl virus can broadly interact with plant receptor-like kinases. Viruses. 11, 1009. doi: 10.3390/v11111009. PMID: 31683645 PMC6893482

[B25] Gill U. Scott J. W. Shekasteband R. Ogundiwin E. Schuit C. Francis D. M. . (2019). Ty-6, a major begomovirus resistance gene on chromosome 10, is effective against Tomato yellow leaf curl virus and Tomato mottle virus. Theor. Appl. Genet. 132, 1543–1554. doi: 10.1007/s00122-019-03298-0. PMID: 30758531 PMC6476845

[B26] Glick M. Levy Y. Gafni Y. (2009). The viral etiology of tomato yellow leaf curl disease – A Review. Plant Prot. Sci. 45, 81–97. doi: 10.17221/26/2009-PPS. PMID: 41862420

[B27] Goncalves L. S. A. Rodrigues R. Diz M. S. S. Robaina R. R. do Amaral Júnior A. T. Carvalho A. O. . (2013). Peroxidase is involved in Pepper yellow mosaic virus resistance in Capsicum baccatum var. pendulum. Genet. Mol. Res. 12, 1411–1420. doi: 10.4238/2013.April.26.3, PMID: 23661464

[B28] Gong P. Tan H. Zhao S. Li H. Liu H. Ma Y. . (2021). Geminiviruses encode additional small proteins with specific subcellular localizations and virulence function. Nat. Commun. 12, 4278. doi: 10.1038/s41467-021-24617-4. PMID: 34257307 PMC8277811

[B29] Guo X. Carroll J. W. Macdonald M. R. Goff S. P. Gao G. (2002). The zinc finger antiviral protein directly binds to specific viral mRNAs through the CCCH zinc finger motifs. J. Virol. 78, 12781–12787. doi: 10.1128/jvi.78.23.12781-12787.2004. PMID: 15542630 PMC525010

[B30] Habib A. Powell J. J. Stiller J. Liu M. Shabala S. Zhou M. . (2018). A multiple near isogenic line (multi-NIL) RNA-seq approach to identify candidate genes underpinning QTL. Theor. Appl. Genet. 131, 613–624. doi: 10.1007/s00122-017-3023-0. PMID: 29170790

[B31] Hakmaoui A. Pérez-Bueno M. L. García-Fontana B. Camejo D. Jiménez A. Sevilla F. . (2012). Analysis of the antioxidant response of Nicotiana benthamiana to infection with two strains of pepper mild mottle virus. J. Exp. Bot. 63, 5487–5496. doi: 10.1093/jxb/ers212. PMID: 22915745 PMC3444274

[B32] Hančinský R. Mihálik D. Mrkvová M. Candresse T. Glasa M. (2020). Plant viruses infecting Solanaceae family members in the cultivated and wild environments: a review. Plants 9, 667. doi: 10.3390/plants9050667. PMID: 32466094 PMC7284659

[B33] Hanson P. Green S. K. Kuo G. (2006). Ty-2, a gene on chromosome 11 conditioning geminivirus resistance in tomato. Rep. Tomato Genet. Cooperative. 56, 17–18. Available online at: https://www.readkong.com/page/tomato-genetics-cooperative-report-of-the-volume-56-3860280 (Accessed March 14, 2026).

[B34] Hanssen M. I. Lapidot M. Thomma P. H. J. (2010). Emerging viral diseases of tomato crops. Mol. Plant-Microbe Interact. 23, 539–548. doi: 10.1094/mpmi-23-5-0539. PMID: 20367462

[B35] Hasegawa D. K. Chen W. Zheng Y. Kaur N. Wintermantel W. M. Simmons A. M. . (2018). Comparative transcriptome analysis reveals networks of genes activated in the whitefly, Bemisia tabaci when fed on tomato plants infected with tomato yellow leaf curl virus. Virology 513, 52–64. doi: 10.1016/j.virol.2017.10.008. PMID: 29035786

[B36] Hosmani P. S. Flores-Gonzalez M. Geest H. Maumus F. Bakker L. V. Schijlen E. . (2019). “ An improved de novo assembly and annotation of the tomato reference genome using single-molecule sequencing, Hi-C proximity ligation and optical maps,” in bioRxiv, 767764. doi: 10.1101/767764, PMID:

[B37] Houston K. Tucker M. R. Chowdhury J. Shirley N. Little A. (2016). The plant cell wall: A complex and dynamic structure as revealed by the responses of genes under stress conditions. Front. Plant Sci. 7. doi: 10.3389/fpls.2016.00984. PMID: 27559336 PMC4978735

[B38] Huang Y. Zhang B. L. Sun S. Xing G. M. Wang F. Li M. Y. . (2016). AP2/ERF transcription factors involved in response to tomato yellow leaf curly virus in tomato. Plant Genome 9. doi: 10.3835/plantgenome2015.09.0082, PMID: 27898839

[B39] Hutton S. F. Scott J. W. Schuster D. J. (2012). Recessive resistance to tomato yellow leaf curl virus from the tomato cultivar Tyking is located in the same region as ty-5 on chromosome 4. HortScience 47, 324–324. doi: 10.21273/HORTSCI.47.3.324

[B40] Ji Y. Schuster D. J. Scott J. W. (2007). Ty-3, a begomovirus resistance locus near the tomato yellow leaf curl virus resistance locus Ty-1 on chromosome 6 of tomato. Mol. Breeding. 20, 271. doi: 10.1007/s11032-007-9089-7. PMID: 41859209

[B41] Ji Y. Scott J. W. Schuster D. J. Maxwell D. P. (2009). Molecular mapping of Ty-4, a new tomato yellow leaf curl virus resistance locus on chromosome 3 of tomato. J. Am. Soc Hortic. Sci. 134, 281–288. doi: 10.1007/s11032-007-9089-7. PMID: 41859209

[B42] Jin B. Robertson K. D. (2013). DNA methyltransferases, DNA damage repair, and cancer. Adv. Exp. Med. Biol. 754, 3–29. doi: 10.1007/978-1-4419-9967-2_1. PMID: 22956494 PMC3707278

[B43] Kalloo G. Banerjee M. K. (1990). Transfer of tomato leaf curl virus-resistance from Lycopersicon hirsutum f. glabratum into L. esculentum. Plant Breed. 105, 156–159. doi: 10.1016/b978-0-08-040826-2.50049-7. PMID: 41862359

[B44] Kim D. Paggi J. M. Park C. Bennett C. Salzberg S. L. (2019). Graph-based genome alignment and genotyping with HISAT2 and HISAT-genotype. Nat. Biotechnol. 37, 907–915. doi: 10.1038/s41587-019-0201-4. PMID: 31375807 PMC7605509

[B45] Langmead B. Trapnell C. Pop M. Salzberg S. L. (2009). Ultrafast and memory-efficient alignment of short DNA sequences to the human genome. Genome Biol. 10, R25. doi: 10.1186/gb-2009-10-3-r25. PMID: 19261174 PMC2690996

[B46] Lapidot M. Ben-Joseph R. Cohen L. Machbash Z. Levy D. (2006). Development of a scale for evaluation of tomato yellow leaf curl virus resistance level in tomato plants. Phytopathology. 96, 1404–1408. doi: 10.1094/PHYTO-96-1404. PMID: 18943674

[B47] Lapidot M. Karniel U. Gelbart D. Fogel D. Evenor D. Kutsher Y. . (2015). A novel route controlling begomovirus resistance by the messenger RNA surveillance factor pelota. PloS Genet. 11, e1005538. doi: 10.1371/journal.pgen.1005538. PMID: 26448569 PMC4598160

[B48] Lefeuvre P. Martin D. P. Harkins G. Lemey P. Gray A. J. A. Meredith S. . (2010). The spread of tomato yellow leaf curl virus from the Middle East to the world. PloS Pathog. 6, e1001164. doi: 10.1371/journal.ppat.1001164. PMID: 21060815 PMC2965765

[B49] Li N. Lin Z. Yu P. Zeng Y. Du S. Huang L. J. (2023). The multifarious role of callose and callose synthase in plant development and environment interactions. Front. Plant Sci. 14. doi: 10.3389/fpls.2023.1183402. PMID: 37324665 PMC10264662

[B50] Li Y. Qin L. Zhao J. Muhammad T. Cao H. Li H. . (2017). SlMAPK3 enhances tolerance to tomato yellow leaf curl virus (TYLCV) by regulating salicylic acid and jasmonic acid signaling in tomato (Solanum lycopersicum). PloS One 12, e0172466. doi: 10.1371/journal.pone.0172466. PMID: 28222174 PMC5319765

[B51] Li X. Zhou X. Li F. (2025). Pelota: A double-edged sword in virus infection. PloS Pathog. 21, e1013328. doi: 10.1371/journal.ppat.1013328, PMID: 40638599 PMC12244479

[B52] Lin C. W. Su M. H. Lin Y. T. Chung C. H. Ku H. M. (2015). Functional characterization of Cucumis metuliferus proteinase inhibitor gene (CmSPI) in potyviruses resistance. Viruses 7, 3816–3834. doi: 10.3390/v7072799. PMID: 26184285 PMC4517128

[B53] Ling K.-S. Simmons A. M. Hassell R. L. Keinath A. P. Polston J. E. (2006). First report of tomato yellow leaf curl virus in South Carolina. Plant Dis. 90, 379. doi: 10.1094/pd-90-0379c. PMID: 30786578

[B54] Liu H. Chang Z. Zhao S. Gong P. Zhang M. Lozano-Durán R. . (2023). Functional identification of a novel C7 protein of tomato yellow leaf curl virus. Virology 585, 117–126. doi: 10.1016/j.virol.2023.05.011. PMID: 37331112

[B55] Liu Y. Schiff M. Dinesh-Kumar S. P. (2004). Involvement of MEK1 MAPKK, NTF6 MAPK, WRKY/MYB transcription factors, COI1 and CTR1 in N-mediated resistance to tobacco mosaic virus. Plant J. 38, 800–809. doi: 10.1111/j.1365-313x.2004.02085.x. PMID: 15144381

[B56] Love M. I. Huber W. Anders S. (2014). Moderated estimation of fold change and dispersion for RNA-seq data with DESeq2. Genome Biol. 15, 550. doi: 10.1186/s13059-014-0550-8. PMID: 25516281 PMC4302049

[B57] Ma N. Sun P. Li Z.-Y. Zhang F.-J. Wang X.-F. You C.-X. . (2024). Plant disease resistance outputs regulated by AP2/ERF transcription factor family. Stress Biol. 4, 2. doi: 10.1007/s44154-023-00140-y. PMID: 38163824 PMC10758382

[B58] McHale L. Tan X. Koehl P. Michelmore R. W. (2006). Plant NBS-LRR proteins: adaptable guards. Genome Biol. 7, 212. doi: 10.1186/gb-2006-7-4-212. PMID: 16677430 PMC1557992

[B59] Moffett P. Farnham G. Peart J. Baulcombe D. C. (2002). Interaction between domains of a plant NBS-LRR protein in disease resistance-related cell death. EMBO J. 21, 4511–4519. doi: 10.1093/emboj/cdf453. PMID: 12198153 PMC126192

[B60] Montasser M. S. Al-Own F. D. Haneif A. M. Afzal M. (2012). Effect of Tomato yellow leaf curl bigeminivirus (TYLCV) infection on tomato cell ultrastructure and physiology. Can. J. Plant Pathol. 34, 114–125. doi: 10.1080/07060661.2012.661767. PMID: 41858497

[B61] Navas-Castillo J. Fiallo-Olive E. Sanchez-Campos S. (2011). Emerging virus diseases transmitted by whiteflies. Annu. Rev. Phytopathol. 49, 219–248. doi: 10.1146/annurev-phyto-072910-095235. PMID: 21568700

[B62] Nouraei S. Mia M. S. Liu H. Turner N. C. Yan G. (2022). Transcriptome analyses of near isogenic lines reveal putative drought tolerance controlling genes in wheat. Front. Plant Sci. 13. doi: 10.3389/fpls.2022.857829. PMID: 35422827 PMC9005202

[B63] Padmanabhan C. Ma Q. Shekasteband R. Stewart K. S. Hutton S. F. Scott J. W. . (2019). Comprehensive transcriptome analysis and functional characterization of PR-5 for its involvement in tomato Sw-7 resistance to tomato spotted wilt tospovirus. Sci. Rep. 9, 7673. doi: 10.1038/s41598-019-44100-x. PMID: 31114006 PMC6529424

[B64] Padmanabhan C. Zheng Y. Shamimuzzaman M. Wilson J. R. Gilliard A. Fei Z. . (2022). The tomato yellow leaf curl virus C4 protein alters the expression of plant developmental genes correlating to leaf upward cupping phenotype in tomato. PLoS ONE 17 (5), e0257936. doi: 10.1371/journal.pone.0257936, PMID: 35551312 PMC9098041

[B65] Pico B. Diez Maria J. Nuez F. (1996). Viral diseases causing the greatest economic losses to the tomato crop. II: The tomato yellow leaf curl virus—A review. Sci. Hortic. 67, 151–196. doi: 10.1016/S0304-4238(96)00945-4, PMID: 41537088

[B66] Pico B. Sifres A. Elia M. Diez M. J. Nuez F. (2000). Searching for new resistance sources to tomato yellow leaf curl virus within a highly variable wild Lycopersicon genetic pool. Acta Physiol. Plant 22, 344–350. doi: 10.1007/s11738-000-0051-0. PMID: 41859209

[B67] Piedra-Aguilera Á. Jiao C. Luna A. P. Villanueva F. Dabad M. Esteve-Codina . (2019). Integrated single-base resolution maps of transcriptome, sRNAome and methylome of tomato yellow leaf curl virus (TYLCV) in tomato. Sci. Rep. 9, 2863. doi: 10.1038/s41598-019-39239-6. PMID: 30814535 PMC6393547

[B68] Pikaard C. S. (2013). Methylating the DNA of the most repressed: special access required. Molecular Cell 49, 1021–1022. doi: 10.1016/j.molcel.2013.03.013. PMID: 23541038 PMC3641553

[B69] Prasad A. Sharma N. Hari-Gowthem G. Muthamilarasan M. Prasad M. (2020). Tomato yellow leaf curl virus: impact, challenges, and management. Trends Plant Sci. 25, 897–911. doi: 10.1016/j.tplants.2020.03.015. PMID: 32371058

[B70] Quast C. Pruesse E. Yilmaz P. Gerken J. Schweer T. Yarza P. . (2013). The SILVA ribosomal RNA gene database project: improved data processing and web-based tools. Nucl. Acids Res. 41, D590–D596. doi: 10.1093/nar/gks1219. PMID: 23193283 PMC3531112

[B71] Radwan D. E. Fayez K. A. Mahmoud S. Y. Hamad A. Lu G. (2007). Physiological and metabolic changes of Cucurbita pepo leaves in response to Zucchini yellow mosaic virus (ZYMV) infection and salicylic acid treatments. Plant Physiol. Biochem. 45, 480–489. doi: 10.1016/j.plaphy.2007.03.002. PMID: 17466528

[B72] R Core Team (2018). R: A language and environment for statistical computing (Vienna: R Foundation for Statistical Computing). Available online at: https://www.R-project.org (Accessed March 14, 2026).

[B73] Ren Y. Tao X. Li D. Yang X. Zhou X. (2022). ty-5 confers broad-spectrum resistance to geminiviruses. Viruses 14, 1804. doi: 10.3390/v14081804. PMID: 36016426 PMC9415776

[B74] Sandelin A. Alkema W. Engström P. Wasserman W. W. Lenhard B. (2004). JASPAR: an open-access database for eukaryotic transcription factor binding profiles. Nucleic Acids Res. 32, D91–D94. doi: 10.1093/nar/gkh012. PMID: 14681366 PMC308747

[B75] Scholthof K.-B. Adkins S. Czosnek H. Palukaitis P. Jacquot E. Hohn T. . (2011). Top 10 plant viruses in molecular plant pathology. Mol. Plant Pathol. 12, 938–954. doi: 10.1111/j.1364-3703.2011.00752.x. PMID: 22017770 PMC6640423

[B76] Shen W. Dallas M. B. Goshe M. B. Hanley-Bowdoin L. (2014). SnRK1 phosphorylation of AL2 delays Cabbage leaf curl virus infection in Arabidopsis. J. Virol. 88, 10598–10612. doi: 10.1128/JVI.00761-14. PMID: 24990996 PMC4178870

[B77] Shen X. Gill U. Arens M. Yan Z. Bai Y. Hutton S. F. . (2025). The tomato gene Ty-6, encoding DNA polymerase delta subunit 1, confers broad resistance to Geminiviruses. Theor. Appl. Genet. 138, 22. doi: 10.1007/s00122-024-04803-w. PMID: 39775891 PMC11711579

[B78] Shen X. Yan Z. Wang X. Wang Y. Arens M. Du Y. . (2020). The NLR protein encoded by the resistance gene Ty-2 is triggered by the replication-associated protein Rep/C1 of Tomato yellow leaf curl virus. Front. Plant Sci. 11, 545306. doi: 10.3389/fpls.2020.545306, PMID: 33013967 PMC7511541

[B79] Su Z.-L. Li A.-M. Wang M. Qin C.-X. Pan Y.-Q. Liao F. . (2025). The role of AP2/ERF transcription factors in plant responses to biotic stress. Int. J. Mol. Sci. 26, 4921. doi: 10.3390/ijms26104921. PMID: 40430060 PMC12112388

[B80] Szadeczky-Kardoss I. Csorba T. Auber A. Schamberger A. Nyiko T. Taller J. . (2018). The nonstop decay and the RNA silencing systems operate cooperatively in plants. Nucleic Acids Res. 46, 4632–4648. doi: 10.1093/nar/gky279, PMID: 29672715 PMC5961432

[B81] Tang D. Wang G. Zhou J. M. (2017). Receptor kinases in plant-pathogen interactions: more than pattern recognition. Plant Cell 29, 618–637. doi: 10.1105/tpc.16.00891. PMID: 28302675 PMC5435430

[B82] Ueki S. Citovsky V. (2002). The systemic movement of a tobamovirus is inhibited by a cadmium-ion-induced glycine-rich protein. Nat. Cell Biol. 4, 478–486. doi: 10.1038/ncb806. PMID: 12055637

[B83] Ueki S. Citovsky V. (2005). Identification of an interactor of cadmium ion-induced glycine-rich protein involved in regulation of callose levels in plant vasculature. Proc. Natl. Acad. Sci. U.S.A. 102, 12089–12094. doi: 10.1073/pnas.0505927102. PMID: 16103368 PMC1189354

[B84] Van Eck J. Keen P. Tjahjadi M. (2019). “ Agrobacterium tumefaciens-mediated transformation of tomato,” in Transgenic plants, vol. 1864 . Eds. Kumar S. Barone P. Smith M. ( Humana Press, New York, NY). doi: 10.1007/978-1-4939-8778-8_16, PMID: 30415340

[B85] Verlaan M. G. Hutton S. F. Ibrahem R. M. Kormelink R. Visser R. G. F. Scott J. W. . (2013). The tomato yellow leaf curl virus resistance genes Ty-1 and Ty-3 are allelic and code for DFDGD-Class RNA–Dependent RNA polymerases. PloS Genet. 9, e1003399. doi: 10.1371/journal.pgen.1003399. PMID: 23555305 PMC3610679

[B86] Vidavski F. Czosnek H. Gazit S. Levy D. Lapidot M. (2008). Pyramiding of genes conferring resistance to tomato yellow leaf curl virus from different wild tomato species. Plant Breed. 127, 625–631. doi: 10.1111/j.1439-0523.2008.01556.x. PMID: 41858021

[B87] Wang J. Chen T. Zhang Z. Song M. Shen T. Wang X. . (2025). Remodelling autoactive NLRs for broad spectrum immunity in plants. Nature 645, 737–745. doi: 10.1038/s41586-025-09252-z. PMID: 40670781

[B88] Wang P. Sun S. Liu K. Peng R. Li N. Hu B. . (2022). Physiological and transcriptomic analyses revealed gene networks involved in heightened resistance against tomato yellow leaf curl virus infection in salicylic acid and jasmonic acid treated tomato plants. Front. Microbiol. 13. doi: 10.3389/fmicb.2022.970139. PMID: 36187991 PMC9515787

[B89] Yamaguchi H. Ohnishi J. Saito A. Ohyama A. Nunome T. Miyatake K. . (2018). An NB-LRR gene, TYNBS1, is responsible for resistance mediated by the Ty-2 begomovirus resistance locus of tomato. Theor. Appl. Genet. 131, 1345–1362. doi: 10.1007/s00122-018-3082-x. PMID: 29532116

[B90] Yan Z. Wolters A. M. A. Navas-Castillo J. Bai Y. (2021). The global dimension of tomato yellow leaf curl disease: current status and breeding perspectives. Microorganisms 9, 740. doi: 10.3390/microorganisms9040740. PMID: 33916319 PMC8066563

[B91] Zamir D. Ekstein-Michelson I. Zakay Y. Navot N. Zeidan M. Sarfatti M. . (1994). Mapping and introgression of a tomato yellow leaf curl virus tolerance gene, TY-1. Theor. Appl. Genet. 88, 141–146. doi: 10.1007/bf00225889. PMID: 24185918

[B92] Zarei A. Körbes A. P. Younessi P. Montiel G. Champion A. Memelink J. (2011). Two GCC boxes and AP2/ERF-domain transcription factor ORA59 in jasmonate/ethylene-mediated activation of the PDF1.2 promoter in Arabidopsis. Plant Mol. Biol. 75, 321–331. doi: 10.1007/s11103-010-9728-y. PMID: 21246258 PMC3044237

[B93] Zhang C. Wang D. Li Y. Wang Z. Wu Z. Zhang Q. . (2024). Gibberellin positively regulates tomato resistance to tomato yellow leaf curl virus (TYLCV). Plants 13, 1277. doi: 10.3390/plants13091277. PMID: 38732492 PMC11085062

[B94] Zhao S. Gong P. Ren Y. Liu H. Li H. Li F. . (2022). The novel C5 protein from tomato yellow leaf curl virus is a virulence factor and suppressor of gene silencing. Stress Biol. 2, 19. doi: 10.1007/s44154-022-00044-3. PMID: 37676365 PMC10442036

[B95] Zheng Y. Jiao C. Sun H. Rosli H. G. Pombo M. A. Zhang P. . (2016). iTAK: a program for genome-wide prediction and classification of plant transcription factors, transcriptional regulators, and protein kinases. Mol. Plant 9, 1667–1670. doi: 10.1016/j.molp.2016.09.014. PMID: 27717919

[B96] Zhong S. Joung J.-G. Zheng Y. Chen Y. Liu B. Shao Y. . (2011). High-throughput illumina strand-specific RNA sequencing library preparation. Cold Spring Harb. Protoc. 2011 (8), 940–949. doi: 10.1101/pdb.prot5652. PMID: 21807852

[B97] Zhu T. Zhou X. Zhang J.-L. Zhang W.-H. Zhang L.-P. You C.-X. . (2022). Ethylene-induced NbMYB4L is involved in resistance against tobacco mosaic virus in Nicotiana benthamiana. Mol. Plant Pathol. 23, 16–31. doi: 10.1111/mpp.13139. PMID: 34633738 PMC8659562

