# Supplementary material for: Elucidation of the ty-5 resistance network in tomato against tomato yellow leaf curl virus reveals the involvement of AP2/ERF gene
Source: Front Plant Sci. 2026 Apr 15;17:1788099. doi: 10.3389/fpls.2026.1788099 (PMC13124728; doi:10.3389/fpls.2026.1788099)
Supplement: Supplementary file 1 [file Presentation1.pptx]

## Slide 1
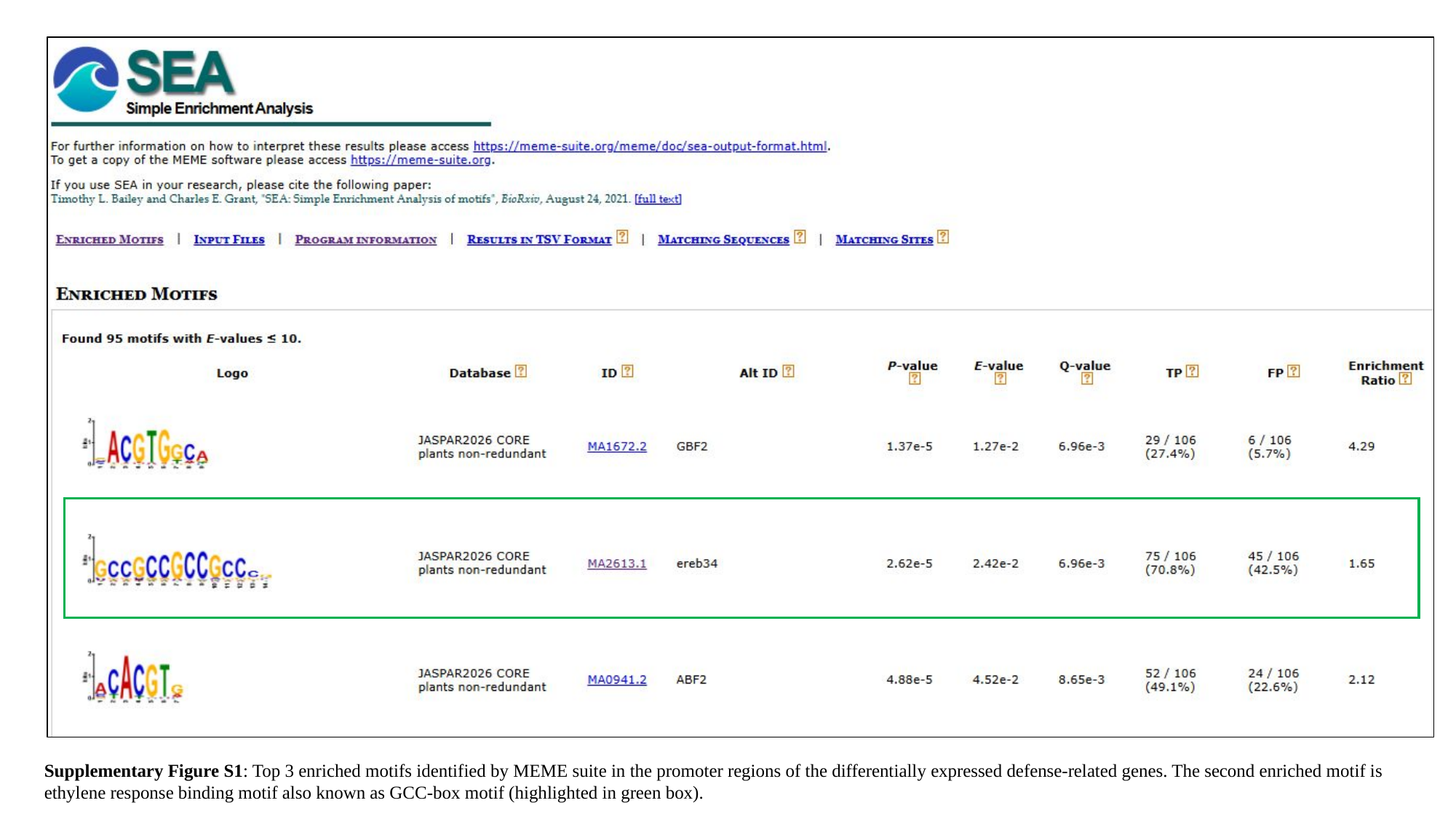

Supplementary Figure S1: Top 3 enriched motifs identified by MEME suite in the promoter regions of the differentially expressed defense-related genes. The second enriched motif is ethylene response binding motif also known as GCC-box motif (highlighted in green box).
