# Supplementary material for: Elucidation of the ty-5 resistance network in tomato against tomato yellow leaf curl virus reveals the involvement of AP2/ERF gene
Source: Front Plant Sci. 2026 Apr 15;17:1788099. doi: 10.3389/fpls.2026.1788099 (PMC13124728; doi:10.3389/fpls.2026.1788099)
Supplement: Supplementary file 2 [file Presentation2.pptx]

## Slide 1
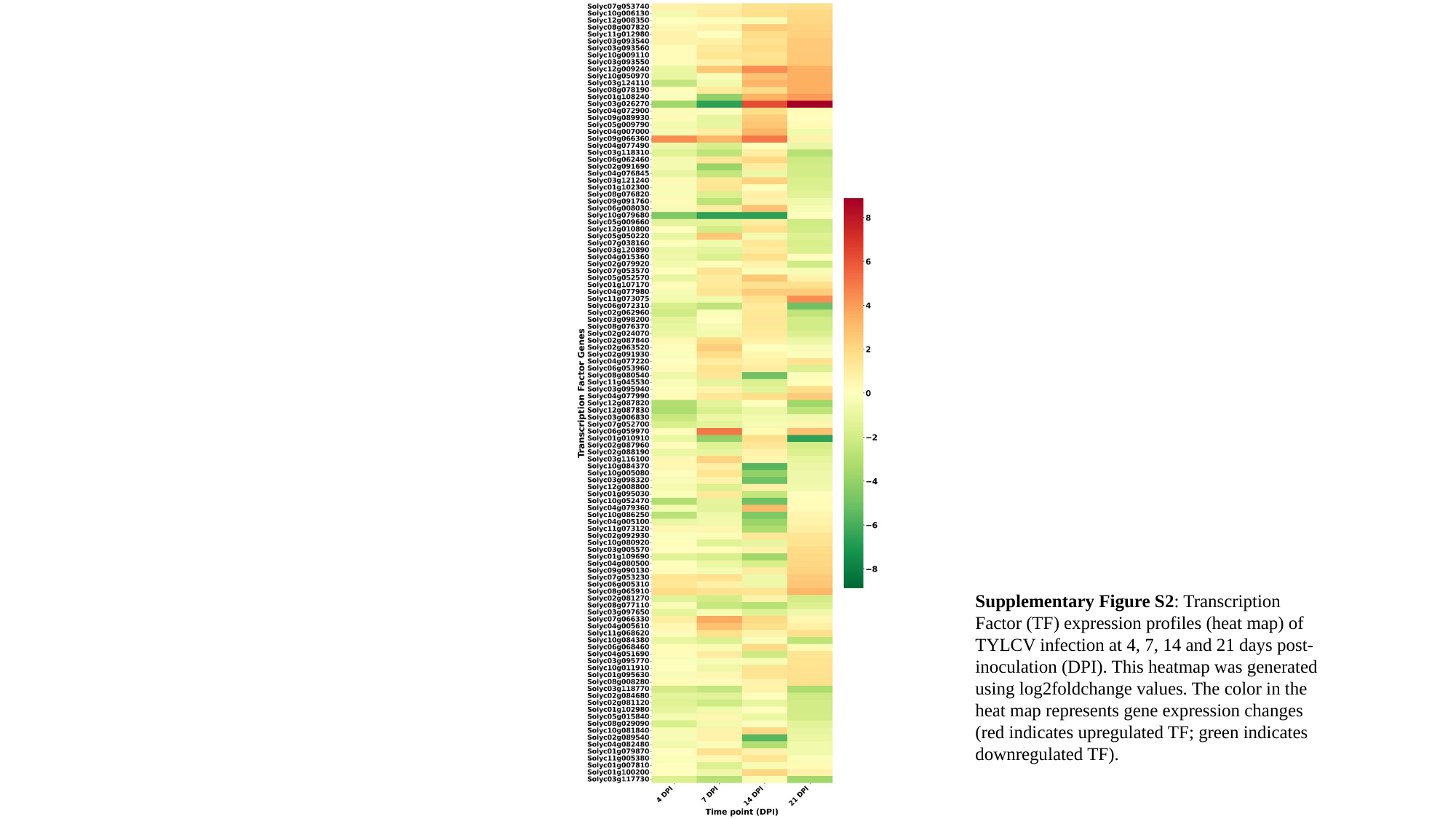

Supplementary Figure S2: Transcription Factor (TF) expression profiles (heat map) of TYLCV infection at 4, 7, 14 and 21 days post-inoculation (DPI). This heatmap was generated using log2foldchange values. The color in the heat map represents gene expression changes (red indicates upregulated TF; green indicates downregulated TF).
